# Superconducting Lithium Hydride in a Chemical Capacitor Setup: A Theoretical Study

**DOI:** 10.1002/cphc.202500013

**Published:** 2025-05-27

**Authors:** Piotr G. Szudlarek, Christopher Renskers, Elena Roxana Margine, Wojciech Grochala

**Affiliations:** ^1^ Center of New Technologies University of Warsaw Zwirki i Wigury 93 02089 Warsaw Poland; ^2^ Department of Physics, Applied Physics, and Astronomy Binghamton University‐SUNY Binghamton NY 13902 USA

**Keywords:** Bardeen–Cooper–Schrieffer theory, density functional theory, hydrides, metallization, superconductivity

## Abstract

Metallization of the ionic hydride LiH has never been achieved experimentally, even under high external pressure. Herein, a novel “chemical capacitor” setup to facilitate its metallization under ambient pressure conditions is applied. The findings reveal that a single layer of this material can withstand doping levels up to an impressive 0.61 holes per H atom without structural collapse, as demonstrated in the ZrC | LiH | ZrC system. Additionally, the electron–phonon coupling strength (*λ*) reaches a remarkable value of 2.1 in the TiO | LiH | TiO system, indicative of the strong coupling regime. Superconductivity calculations further predict a maximum critical temperature ($T_{c}$) of 17.5 K for 0.31‐hole‐doped LiH with (LiBaF_3_)_2_ as surrounding support layers in the absence of external pressure.

## Graphical abstract

Metallization of bulk lithium hydride has never been achieved through pressure alone. Single‐layer lithium hydride sandwiched between two support layers may resist hole doping up to substantial levels, forming a two‐dimensional metal (Fermi surface shown). This system is predicted to exhibit superconductivity with *T*_c_ values up to 17.5 K under ambient pressure conditions.

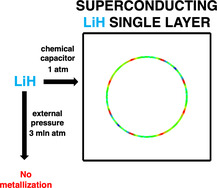

## 1 Introduction

The pursuit of high‐temperature superconductors has been a central focus in condensed matter and materials physics since the discovery of superconductivity in 1911. The Bardeen–Cooper–Schrieffer theory of conventional superconductivity predicts that materials based on light elements might exhibit high critical temperatures ($T_{c}$),^[^ ^1^ ^]^ and, in 1968, N. Ashcroft was the first to suggest the possibility of room‐temperature superconductivity in metallic hydrogen.^[^ ^2^ ^]^ Subsequent efforts to realize superconductivity in metallic hydrogen^[^ ^2^, ^3^, ^4^, ^5^ ^]^ and metal hydrides^[^ ^6^, ^7^, ^8^, ^9^, ^10^ ^]^ have led to the discovery of several hydrogen‐rich superconductors with remarkable $T_{c}$ values approaching room temperature, namely, H_3_S (203 K at 155 GPa),^[^ ^6^ ^]^ LaH_*x*−10_ (260 K at 188 GPa),^[^ ^7^, ^8^ ^]^ CaH_6_ (210 K at 160 GPa),^[^ ^9^ ^]^ and YH_*x*_ (243 K at 201 GPa).^[^ ^10^ ^]^ Importantly, several of these materials were first predicted through theoretical calculations prior to their experimental synthesis,^[^ ^11^, ^12^, ^13^, ^14^ ^]^ and, additionally, further valuable predictions were made using density functional theory (DFT).^[^ ^15^, ^16^, ^17^, ^18^ ^]^ Unfortunately, all these systems require extremely high pressures for metallization, exceeding 100 GPa, and the volumes of their superconducting phases are typically less than 10^−5^ mm^3^, rendering their practical application unfeasible. Therefore, Th_4_H_15_ (8.3 K), PdH_*x*−1_ (9.0 K), and (Pd,Ag)H_*x*−1_ (16 K) stand out as record‐high $T_{c}$ metal hydrides at ambient pressure.^[^ ^19^, ^20^, ^21^ ^]^

Among light‐element hydrides, LiH, which crystallizes in the NaCl structure with a band gap of nearly 5.0 eV,^[^ ^22^ ^]^ is a stable prototypical ionic hydride that can be melted without thermal decomposition.^[^ ^23^ ^]^ Calculations predict that pressure‐induced metallization and a structural transformation to the CsCl phase occur simultaneously at ≈330 GPa.^[^ ^24^ ^]^ Notably, this pressure exceeds that required for the metallization of the aforementioned hydrogen‐rich materials, and pressure‐induced metallization in LiH has never been achieved experimentally.

**Figure 1:**
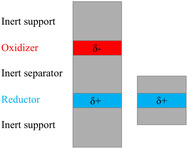
General scheme of a chemical capacitor (left)^[^ ^25^ ^]^ and truncated model (right) used here for superconductivity calculations in hydrides.

Recently, we have taken a different approach to induce metallization in large bandgap insulators.^[^ ^25^ ^]^ Advances in nanotechnology now enable the fabrication of atomic structures one atomic layer at a time on various supports. Techniques such as molecular beam epitaxy, chemical vapor deposition, co‐sputtering, and laser ablation offer feasible routes to realize the systems proposed in ref. [25] and in the present study. For example, previously theorized superconducting Fe–B compounds^[^ ^26^ ^]^ were fabricated by co‐sputtering for a composition spread where reproducible superconductivity was found for an average composition of FeB 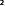 with a $T_{c}$ around 4 K.^[^ ^27^ ^]^ The placement of one or more layers of a strong oxidizer in the vicinity of a reductor, as illustrated in **Figure** **1**, can lead to a substantial charge transfer between the two. For instance, when a single layer of AgF_2_ (oxidizer), with a bandgap of ≈3.6 eV,^[^ ^28^ ^]^ is separated from a single layer of LiH (reductor), with a direct bandgap of 5.0 eV, by a few layers of an inert insulator such as RbMgF_3_, the charge transferred between the two single layers, *δ*, can reach up to 0.67 holes (h^+^) per H atom.^[^ ^25^ ^]^ The magnitude of *δ* can be controlled by varying the thickness of the insulating separator between the oxidizer and the reductor. Such a simple setup, called a chemical capacitor (CC),^[^ ^29^ ^]^ permits mutual metallization of both the electron‐rich and electron‐poor layers.

In this study, we investigate systems containing LiH that undergo substantial hole doping in a CC setup. Our objective is to verify, using DFT calculations, the doping limits ($\delta_{\text{max}}$) in these diverse systems, i.e., the maximum density of holes that can be introduced into these insulators without leading to the appearance of imaginary phonons. Additionally, we aim to predict the critical temperatures they could achieve using the McMillan–Allen–Dynes formula,^[^ ^30^, ^31^ ^]^ derived from the Eliashberg theory.^[^ ^32^ ^]^ Since calculations of superconducting properties for systems with more than a few dozen atoms in the unit cell remain prohibitively expensive, we have been forced to truncate the complex setup to a single layer of reductor and one or two surrounding support/separator layers, as shown in Figure 1. In the absence of an oxidizer, we simply vary the amount of holes in the hydride layer, compensating with a uniform jellium background.^[^ ^33^ ^]^ Detailed information on the methodology and calculations performed with the Quantum ESPRESSO suite^[^ ^34^, ^35^ ^]^ along with full structural information for all systems at δ=0 and $\delta_{\text{max}}$ is provided in Section S1 and S5, Supporting Information,^[^ ^36^ ^]^ respectively.

## 2 Results and Discussion

**Figure 2:**
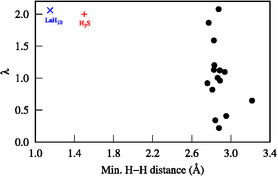
Dependence of the electron–phonon coupling strength (*λ*) as a function of the shortest H–H distance. Data for LaH_10_ and H_3_S were taken from refs. [37, 38, 44, 45] respectively.

Results for the 15 insulator | hydride | insulator two‐dimensional (2D) systems investigated in this work are summarized in **Table** **1**. Electronic band structures, phonon spectra, and Eliashberg spectral functions with integrated electron–phonon coupling strength at δ=0 and $\delta_{\text{max}}$ are shown in Section S2 and S3, Supporting Information.^[^ ^36^ ^]^ The studied systems adopt the tetragonal crystal structure with lattice constant, *a*, ranging from 2.78 to 4.94 Å. Support systems were chosen such that the lattice mismatch does not exceed ±10%, one of the criteria for epitaxy, with the exception of ZrC as seen by the $a_{\text{sep}}/a_{\text{LiH}}$ column in Table 1. The investigated structures exhibit the shortest H–H distances between 2.75 and 3.50 Å, as illustrated in **Figure** **2**, and these H–H distances are considerably longer than those reported for H_3_S at 155 GPa (≈1.5 Å)^[^ ^37^ ^]^ or LaH_10_ at 210 GPa (1.1–1.2 Å).^[^ ^38^ ^]^ The longer H–H contacts in our systems result in a twofold reduction in the maximum phonon frequency.

**Table 1:** Results for the 15 monolayer systems investigated in this study: the tetragonal lattice constant *a*, the lattice mismatch between the separator layer and LiH ($a_{\text{sep}}/a_{\text{LiH}}$), the smallest distance between any two hydrogen atoms (H–H_min_), the maximum doping achieved before dynamic instability $\delta_{\text{max}}$, the density of states (DOS) at the Fermi level $N(E_{F})$, the integrated electron–phonon coupling strength *λ*, the logarithmic average phonon frequency $\omega_{\text{log}}$, and the superconducting critical temperature $T_{c}$ calculated with the McMillan–Allen–Dynes formula with a coulomb potential $\mu^{*}=0.1$.

System			*a* [Å]	$a_{\text{sep}}/a_{\text{LiH}}$	H–H_min_ [Å]	$\delta_{\text{max}}$ [$h^{+}$/H]	$N(E_{F})$ [States eV u.c.^−1^]	$\omega_{\text{log}}$ [meV]	*λ*	$T_{c}$ [K]
Support	Reductor	Support								
VN	LiH	VN	2.76	1.03	2.76	0.32	4.37	19.1	0.92	13.6
LiF	LiH	LiF	2.78	1.01	2.78	0.31	0.50	10.7	1.85	17.0
(LiF)_2_	LiH	(LiF)_2_	2.81	1.01	2.81	0.29	0.50	20.7	0.82	11.6
TiO	LiH	TiO	2.87	1.07	2.87	0.47	3.84	6.6	2.08	11.1
MgO	LiH	MgO	2.88	1.06	2.88	0.44	4.94	27.6	0.22	–
TiN	LiH	TiN	2.89	1.06	2.89	0.59	3.08	17.6	0.96	13.3
MoC	LiH	MoC	2.94	1.09	2.94	0.15	3.69	16.9	1.10	15.7
TiC	LiH	TiC	2.95	1.08	2.95	0.40	0.60	17.8	0.41	0.9
ZrC	LiH	ZrC	3.22	1.15	3.22	0.61	1.35	10.2	0.65	3.1
KMgF_3_	LiH	KMgF_3_	4.00	1.01	2.83	0.25	1.10	6.8	1.59	9.7
(KMgF_3_)_2_	LiH	(KMgF_3_)_2_	4.02	1.01	2.84	0.20	1.16	34.1	0.34	0.7
LiBaF 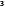	LiH	LiBaF 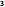	4.00	1.01	2.83	0.31	1.08	17.6	1.13	17.0
(LiBaF 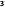 ) 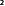	LiH	(LiBaF 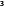 ) 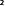	4.00	1.01	2.83	0.31	1.12	18.7	1.20	17.5
RbMgF 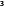	LiH	RbMgF 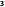	4.05	1.03	2.87	0.25	1.23	11.9	1.00	10.4
(RbMgF 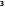 ) 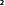	LiH	(RbMgF 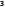 ) 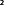	4.08	1.03	2.89	0.27	1.40	11.1	1.12	10.5

**Figure 3:**
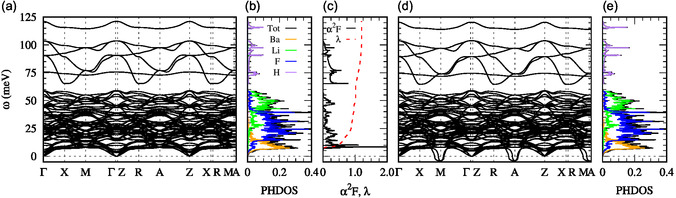
a–c) Phonon dispersion, phonon DOS, Eliashberg spectral function $\alpha^{2}$F, and integrated electron–phonon coupling strength *λ* for (LiBaF_3_)_2_ | LiH | (LiBaF_3_)_2_ at *δ* = 0.31 h^+^. d,e) Phonon dispersion and phonon DOS for the same system at *δ* = 0.32 h^+^, where the system becomes dynamical unstable. Notice the emergence of imaginary modes at the M and A high‐symmetry points.

The $\delta_{\text{max}}$ values presented in Table 1 highlight significant variations in doping sensitivity across the 15 systems analyzed. Some, such as MoC | LiH | MoC, exhibit phonon instabilities at relatively low doping levels (0.15 h^+^), while others, like ZrC | LiH | ZrC, are highly resilient, with maximum hole doping levels reaching 0.61 h^+^ per H atom despite exhibiting the largest lattice mismatch. The remarkable resistance of these systems to gap‐opening lattice distortions is noteworthy, given that even smaller doping levels of 1/2, 1/3, or 1/4 might suggest facile Peierls distortion.^[^ ^39^ ^]^ For example, a doping level of 1/3 corresponds to a polyhydride formula 1/2 H_2_ + 2 H^−^. Such distortion would inevitably lead to symmetry lowering of the lattice and a concomitant bandgap opening at the Fermi level $\left(E_{F}\right)$. However, all systems preserve the high symmetry of the crystal lattice up to the doping level of $\delta_{\text{max}}$. Although phonon softening is observed as the doping level increases, genuine dynamic instability only occurs beyond $\delta_{\text{max}}$, as demonstrated in **Figure** **3**d for (LiBaF_3_)_2_ | LiH | (LiBaF_3_)_2_. This resistance to structural distortion is associated with the presence of adjacent support layers sandwiching the hydride monolayer.

**Figure 4:**
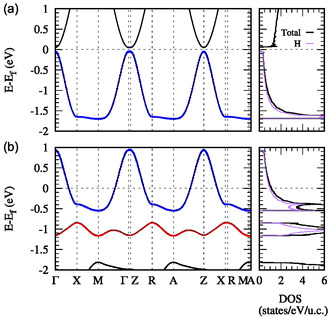
Electronic band structure for (LiBaF_3_)_2_ | LiH | (LiBaF_3_)_2_ at a) *δ* = 0 and b) $\delta_{\text{max}}$ = 0.31 h^+^. H‐1*s* character is shown using circles, blue for one H atom, and red for another in the [Li_2_H_2_] layer. The total DOS is shown in black, and the contribution of the H‐1*s* states to the total DOS is shown in purple.

The heavily doped insulator | hydride | insulator systems exhibit metallic behavior, with the doped band at the Fermi level arising almost entirely from H‐1*s* states, as illustrated in **Figure** **4** for (LiBaF_3_)_2_ | LiH | (LiBaF_3_)_2_. These hydride systems are essentially single‐band metals, containing a 2D metallic hydrogen sublattice. In essence, they represent a long‐sought realization of “metallic hydrogen at ambient pressure”.

Next, we investigate the superconducting properties of the 15 systems. According to the McMillan–Allen–Dynes expression, $T_{c}$ depends linearly on the logarithmic average phonon frequency, $\omega_{\text{log}}$, and exponentially on the integrated electron–phonon coupling strength, *λ* (see Section S1, Supporting Information, for the detailed formula^[^ ^36^ ^]^). While $\omega_{\text{log}}$ reflects the frequencies most effective for superconductivity, weighted by the electron–phonon coupling, *λ* captures how phonons at different frequencies couple to electrons, through the Eliashberg spectral function $\alpha^{2}F(\omega )$. Additionally, $T_{c}$ is directly influenced by the electronic DOS at the Fermi level, as a higher DOS linearly increases *λ*. In our simple one‐band systems, the DOS increases monotonically with the doping level, reaching its maximum at $\delta_{\text{max}}$, as illustrated for (RbMgF_3_)_2_ | LiH | (RbMgF_3_)_2_ in Figure S3, Supporting Information.^[^ ^36^ ^]^ Increased doping, however, can induce phonon softening, which reduces $\omega_{\text{log}}$ but enhances *λ*, as shown for the same system in Figure S4 and S5, Supporting Information.^[^ ^36^ ^]^ Consequently, the net effect on $T_{c}$ is governed by the interplay among these parameters, highlighting the challenges in designing high‐temperature superconductors.

From the analysis of $N(E_{F})$, $\omega_{\text{log}}$, *λ*, and $T_{c}$ at $\delta_{\text{max}}$ in Table 1, we can draw several important observations. The studied systems achieve electronic total DOS values of up to ≈5 states (eV u.c.)^−1^, as demonstrated by MgO | LiH | MgO. These values are comparable to, or even exceed, those of typical metals. For example, a DOS of 0.95 states (eV · u.c.)^−1^ is computed for bulk bcc Li using the same methodology. However, a large DOS does not necessarily result in superconductivity, as found in MgO | LiH | MgO that shows no discernible $T_{c}$. The use of two surrounding support layers, instead of one, appears to either have no impact on superconductivity [e.g., (LiBaF_3_)_2_ | LiH | (LiBaF_3_)_2_ and (RbMgF_3_)_2_ | LiH | (RbMgF_3_)_2_] or to be detrimental [e.g., (LiF)_2_ | LiH | (LiF)_2_ and (KMgF_3_)_2_ | LiH | (KMgF_3_)_2_]. Across the systems, $\lambda_{\text{max}}$ ranges from 0.2 to 2.1. Interestingly, despite not exhibiting any H—H bonding, some of the systems may achieve very large lambda values, exceeding 2, that are comparable to other strongly coupled pressurized hydrides, as seen in Figure 2. This implies that strong electron–phonon coupling might be achievable in noncovalent materials. The upper limit corresponds to strong coupling (*λ* > 1), surpassing that of metallic Pb (*λ* = 1.55).^[^ ^40^ ^]^ As a result, systems with both high DOS and *λ* values yield $T_{c}$ values exceeding 10.0 K. Notably, a maximum $T_{c}$ of 17.5 K is predicted for (LiBaF_3_)_2_ | LiH | (LiBaF_3_)_2_, which surpasses the experimentally observed $T_{c}$ of 16.0 K for (Pd,Ag)H_1−*x*_.^[^ ^21^ ^]^

**Figure 5:**
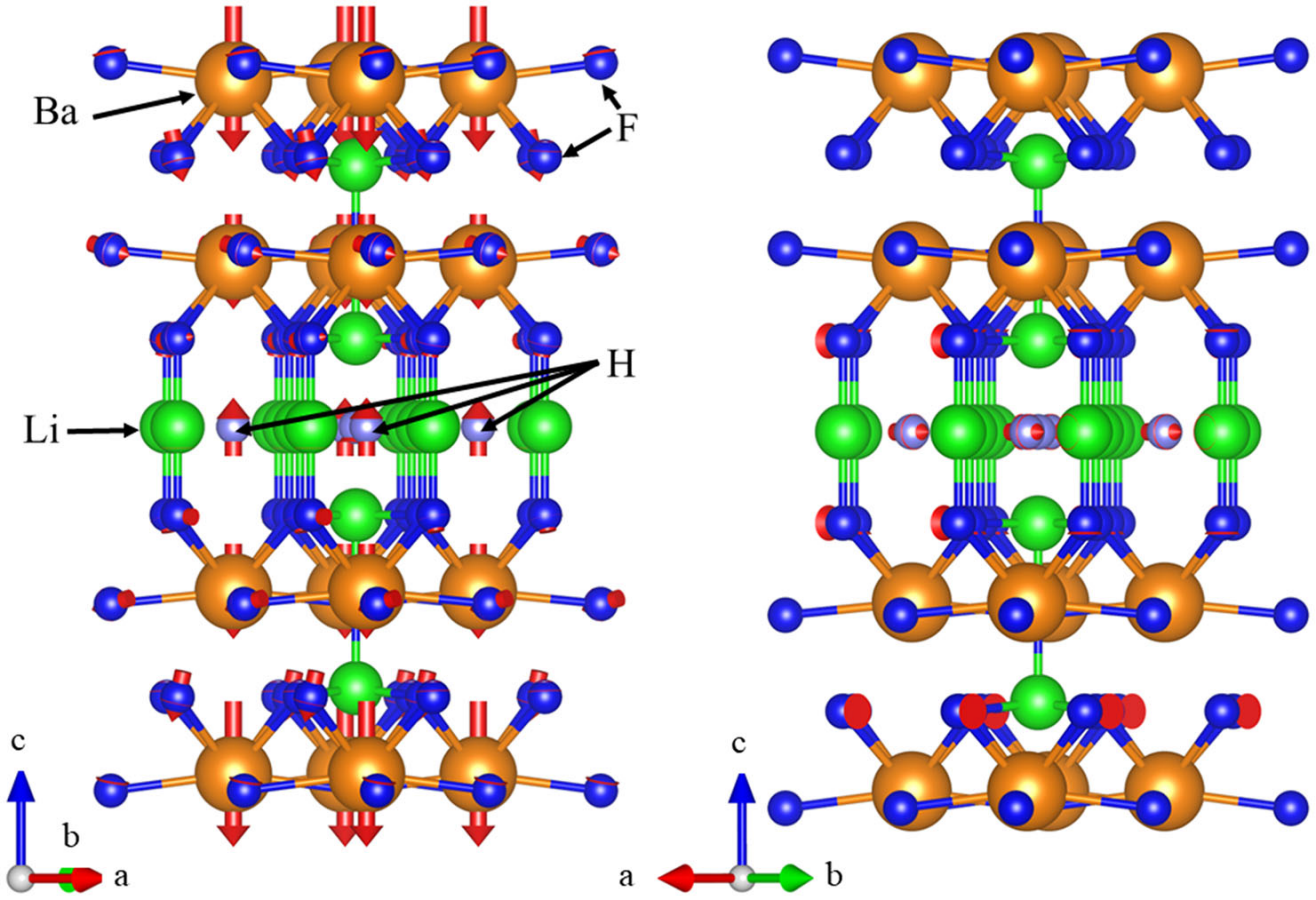
Atomic displacements for two selected phonon modes which show appreciable electron–phonon coupling for (LiBaF_3_)_2_ | LiH | (LiBaF_3_)_2_ at $\delta_{\text{max}}$ = 0.31 h^+^: 8.7 meV (left) and 65 meV (right). The orange atoms correspond to Ba, green to Li, blue to F, and light purple to H.

Next, it is instructive to gain insight into the character of the phonons that couple with the electrons in the studied systems. To illustrate this, we select (LiBaF_3_)_2_ | LiH | (LiBaF_3_)_2_. The presence of the lightest element, H, leads to phonon frequencies as high as 125 meV. It is natural then to expect, similar to the highest *T*_c_ hydrides,^[^ ^37^, ^38^ ^]^ that high‐frequency phonons associated with the motion of H atoms would couple strongly to electrons in what is essentially metallic hydrogen. Surprisingly, however, high‐frequency vibrations of H contribute only 17% of the total integrated *λ*, as shown in Figure 3a–c for (LiBaF_3_)_2_ | LiH | (LiBaF_3_)_2_. Nevertheless, a 65 meV mode at the *Γ* point involving predominantly in‐plane motion of H atoms contributes to the electron–phonon coupling (see **Figure** 3 and **5**). The largest contribution to *λ*, 83%, arises from the motion of the heavy Ba, Li, and F atoms. A notable example of low‐frequency phonons coupling strongly with electrons is the 8.7 meV phonon mode in the (LiBaF_3_)_2_ | LiH | (LiBaF_3_)_2_ system. This mode involves motion of heavy Ba atoms coupled with some out‐of‐plane contribution from H. Similar features are observed in systems featuring RbMgF_3_ sandwich layers, where Rb‐dominated phonons make substantial contributions to *λ*. These findings suggest that seemingly inert sandwiching layers can significantly influence superconductivity in the hydride layer through proximity effects.^[^ ^41^, ^42^ ^]^

## 3 Conclusions

In summary our first‐principles calculations demonstrate that LiH‐based systems can not only withstand high doping levels without structural collapse, but can also achieve superconducting temperatures up to 17.5 K in the absence of external pressure. The stoichiometries and structures explored in this study are simple and rather H‐poor compared to superhydrides such as H_3_S and LaH_10_, while the Periodic Table provides an extensive playground for further explorations.^[^ ^23^ ^]^ The present findings motivate future theoretical investigations in the quest of H‐rich superconducting materials with even higher $T_{c}$ values at ambient conditions.

## Conflict of Interest

The authors declare no conflict of interest.

## Author Contributions

The manuscript was written through contributions of all authors. All authors have given approval to the final version of the manuscript. **Piotr G. Szudlarek** and **Christopher Renskers** contributed equally to this work.

## Supporting information

Supplementary Material

## Data Availability

The data that support the findings of this study are available in the supplementary material of this article.
