# Supplementary material for: Superconducting Lithium Hydride in a Chemical Capacitor Setup: A Theoretical Study
Source: Chemphyschem. 2025 May 27;26(13):e202500013. doi: 10.1002/cphc.202500013 (PMC12225749; doi:10.1002/cphc.202500013)
Supplement: Supplementary file 1 — Supplementary Material [file CPHC-26-e202500013-s001.pdf]

Supplemental Material
Superconducting Lithium Hydride in a Chemical Capacitor Setup – a Theoretical Study

Piotr Szudlarek,[1] Christopher Renskers,[2] Elena R. Margine,[2,*] and Wojciech Grochala[1,†]

[1] *Center of New Technologies, University of Warsaw, Zwirki i Wigury 93, 02089 Warsaw Poland*
[2] *Department of Physics, Applied Physics, and Astronomy, Binghamton University-SUNY, Binghamton, New York 13902, USA*

Contents

S1. Methodology 1

S2. Electronic properties 3

S3. Vibrational and superconducting properties 6

S4. Dependence with doping for $(RbMgF_3)_2$ | LiH | $(RbMgF_3)_2$ 9

S5. Crystal properties at zero and max doping 10

References 43

S1. Methodology

We first built bulk 3D models of the novel "chemical capacitor" setup consisting of the inert support, inert separator, oxidizer, and reductor as shown on the left side in Fig. 1 of the main text. Due to the computational complexity of *ab initio* calculations of such systems, in particular superconductivity, we considered a truncated 2D model that consists only of the inert support, inert separator, and reductor as shown on the right side in Fig. 1 of the main text.

Density functional theory calculations were carried out with the Quantum ESPRESSO (QE) package [3, 4]. We used the Perdew-Burke-Ernzerhof (PBE) [8] generalized gradient approximation and optimized norm-conserving Vanderbilt (ONCV) pseudopotentials from the Pseudo Dojo library [9]. A plane-wave cutoff value of 100 Ry, a Methfessel-Paxton smearing [6] value of 0.01 Ry, and Γ-centered Monkhorst-Pack [7] **k** meshes were used to describe the electronic structure. A large cutoff of 100 Ry was needed to converge the energy for the $RbMgF_3$ | LiH | $RbMgF_3$ system and was subsequently kept for all others for ease and uniformity. Meshes used for each system are listed in Table S1. Since these are 2D materials we only sampled with one point in the out of plane direction, and used a vacuum distance of, at least, 12 Å for all systems to insure no effects from periodicity in the out of plane direction. The atomic positions and in-plane lattice constants were optimized until the total energy was converged with 10^{-6} Ry and the force on each atom was less than 10^{-4} Ry/Å while keeping the vacuum spacing constant.

To study hole doped systems, we employed a jellium model. In this case, we relaxed the atomic positions while holding the lattice parameters fixed at the undoped values. The systems were hole doped until they became dynamically unstable (i.e., the appearance of imaginary phonons). A doping level as high as 0.61 electrons per H was reached in ZrC | LiH | ZrC. The dynamical matrices and electron-phonon (e-ph) matrix elements were computed using density-functional perturbation theory (DFPT) [2] on irreducible sets of regular **q** meshes. For each **k**-point, the e-ph matrix elements were linearly interpolated to a denser **k**-mesh [10] denoted by the 'interpolated **k**-mesh' column in Table S1. Finally, the Eliashberg spectral function ($\alpha^2 F$) was computed for a set of broadenings from 0 to 0.08 Ry [10]. We identified 0.03 Ry as the optimal choice for smearing and estimated the superconducting critical temperature (T_c) using the Allen-Dynes modified McMillan formula [1, 5] with a value of μ^*=0.10.

* rmargine@binghamton.edu
† w.grochala@cent.uw.edu.pl

The equations for the relevant superconducting quantities: Eliashberg spectral function (α^2F), e-ph coupling strength (λ), logarithmic average phonon frequency (ω_{log}), and superconducting critical temperature (T_{c}) are shown below.

$$\alpha^2F(\omega) = \frac{1}{N_{\mathrm{F}}} \sum_{n\mathbf{k},m\mathbf{q},\nu} |g_{nm\nu}(\mathbf{k},\mathbf{q})|^2 \delta(\epsilon_{n\mathbf{k}} - \epsilon_{\mathrm{F}})\delta(\epsilon_{m\mathbf{k}+\mathbf{q}} - \epsilon_{\mathrm{F}})\delta(\hbar\omega - \hbar\omega_{\mathbf{q}\nu}) \tag{1}$$

$$\lambda = 2\int_0^\infty \frac{\alpha^2F(\omega)}{\omega}\mathrm{d}\omega \tag{2}$$

$$\omega_{\mathrm{log}} = \exp\left[\frac{2}{\lambda}\int_0^\infty \mathrm{d}\omega \frac{\alpha^2F(\omega)}{\omega}\log\omega\right] \tag{3}$$

$$k_{\mathrm{B}}T_c = \frac{\hbar\omega_{\mathrm{log}}}{1.2}\exp\left[-\frac{1.04(1+\lambda)}{\lambda - \mu^*(1+0.62\lambda)}\right] \tag{4}$$

In these expressions, n and $\mathbf{k}$ are the band indices and wavevectors for the electronic state $\epsilon_{n\mathbf{k}}$, ν and $\mathbf{q}$ are the branch indices and wavevectors for the phonon of frequency $\omega_{\mathbf{q}\nu}$, ϵ_{F} is the Fermi level, N_{F} is the density of states per spin at the Fermi level, and $g_{nm\nu}(\mathbf{k},\mathbf{q})$ is the screened e-ph matrix element for the scattering between the electronic states $n\mathbf{k}$ and $m\mathbf{k}+\mathbf{q}$ through a phonon of frequency $\omega_{\mathbf{q}\nu}$.

Support	System Reductor	Support	**k**-mesh	**q**-mesh	Interpolated **k**-mesh
VN	LiH	VN	24 × 24 × 1	8 × 8 × 1	120 × 120 × 1
LiF	LiH	LiF	24 × 24 × 1	8 × 8 × 1	120 × 120 × 1
$(LiF)_2$	LiH	$(LiF)_2$	18 × 18 × 1	6 × 6 × 1	90 × 90 × 1
TiO	LiH	TiO	18 × 18 × 1	6 × 6 × 1	90 × 90 × 1
MgO	LiH	MgO	18 × 18 × 1	6 × 6 × 1	90 × 90 × 1
TiN	LiH	TiN	24 × 24 × 1	8 × 8 × 1	120 × 120 × 1
MoC	LiH	MoC	18 × 18 × 1	6 × 6 × 1	90 × 90 × 1
TiC	LiH	TiC	18 × 18 × 1	6 × 6 × 1	90 × 90 × 1
ZrC	LiH	ZrC	18 × 18 × 1	6 × 6 × 1	90 × 90 × 1
$KMgF_3$	LiH	$KMgF_3$	12 × 12 × 1	4 × 4 × 1	60 × 60 × 1
$(KMgF_3)_2$	LiH	$(KMgF_3)_2$	12 × 12 × 1	4 × 4 × 1	60 × 60 × 1
$LiBaF_3$	LiH	$LiBaF_3$	12 × 12 × 1	4 × 4 × 1	60 × 60 × 1
$(LiBaF_3)_2$	LiH	$(LiBaF_3)_2$	12 × 12 × 1	4 × 4 × 1	60 × 60 × 1
$RbMgF_3$	LiH	$RbMgF_3$	12 × 12 × 1	4 × 4 × 1	60 × 60 × 1
$(RbMgF_3)_2$	LiH	$(RbMgF_3)_2$	12 × 12 × 1	4 × 4 × 1	60 × 60 × 1

TABLE S1: Settings used for the calculation of electronic, vibrational, and superconducting properties of the 15 systems studied with Quantum ESPRESSO [3, 4].

S2. Electronic properties

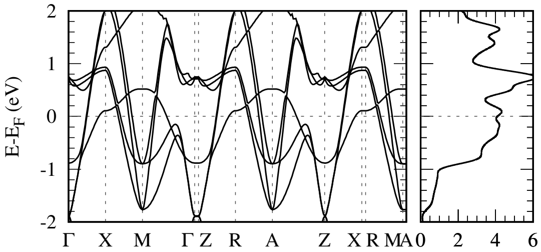

(a) VN | LiH | VN at $\delta_{\max} = 0$ h$^+$

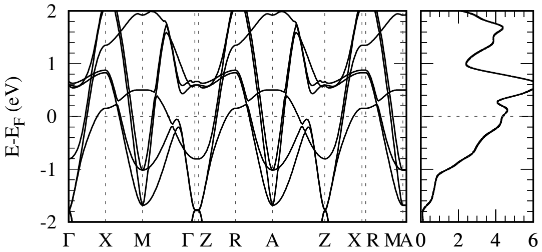

(b) VN | LiH | VN at $\delta_{\max} = 0.32$ h$^+$

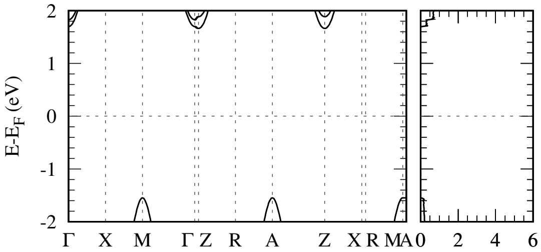

(c) LiF | LiH | LiF at $\delta_{\max} = 0$ h$^+$

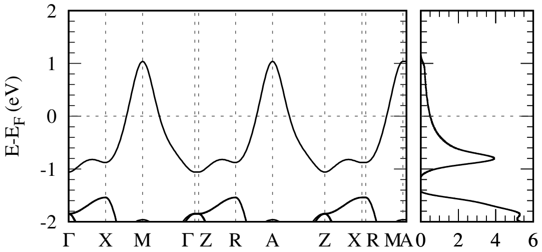

(d) LiF | LiH | LiF at $\delta_{\max} = 0.31$ h$^+$

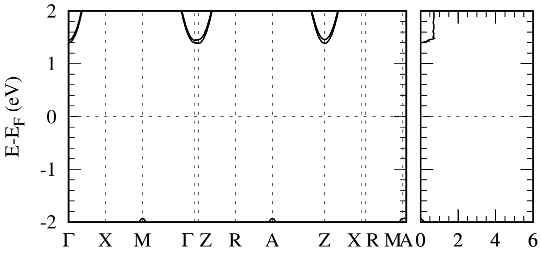

(e) $(LiF)_2$ | LiH | $(LiF)_2$ at $\delta_{\max} = 0$ h$^+$

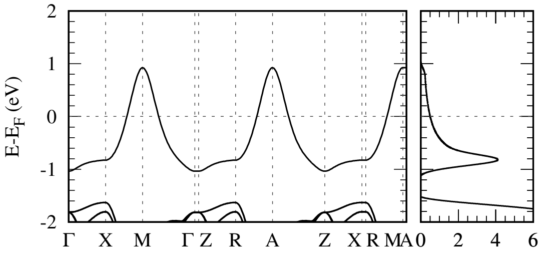

(f) $(LiF)_2$ | LiH | $(LiF)_2$ at $\delta_{\max} = 0.29$ h$^+$

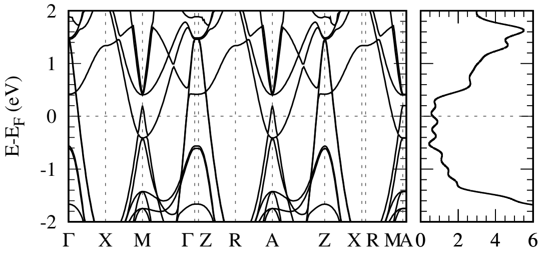

(g) TiO | LiH | TiO at $\delta_{\max} = 0$ h$^+$

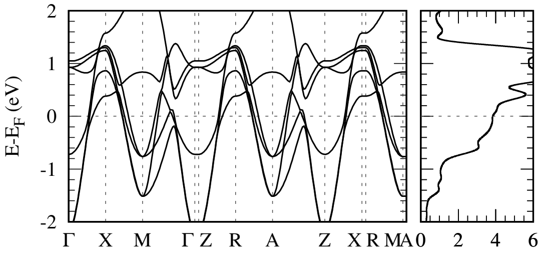

(h) TiO | LiH | TiO at $\delta_{\max} = 0.47$ h$^+$

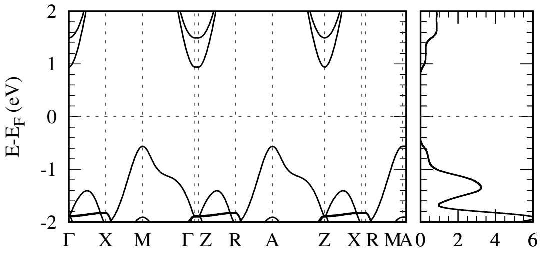

(i) MgO | LiH | MgO at $\delta_{\max} = 0$ h$^+$

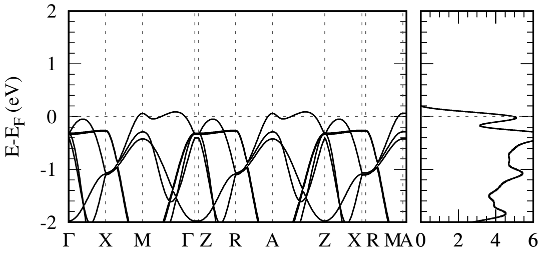

(j) MgO | LiH | MgO at $\delta_{\max} = 0.44$ h$^+$

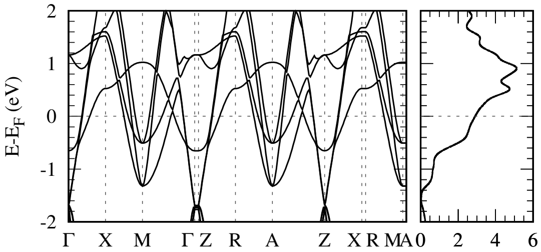

(k) TiN | LiH | TiN at $\delta_{\max} = 0$ h$^+$

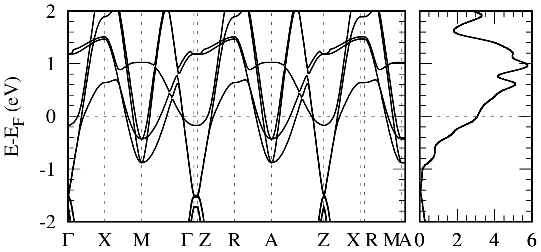

(l) TiN | LiH | TiN at $\delta_{\max} = 0.59$ h$^+$

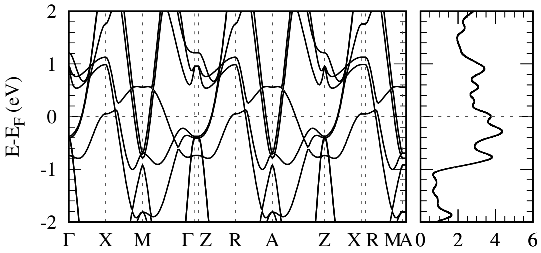

(m) MoC | LiH | MoC at $\delta_{\max} = 0$ h$^+$

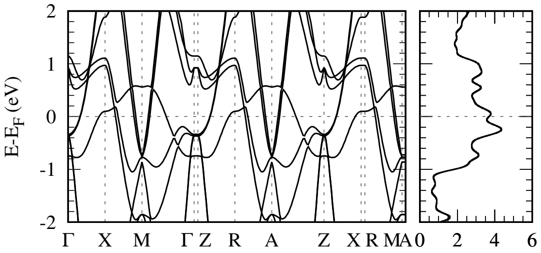

(n) MoC | LiH | MoC at $\delta_{\max} = 0.15$ h$^+$

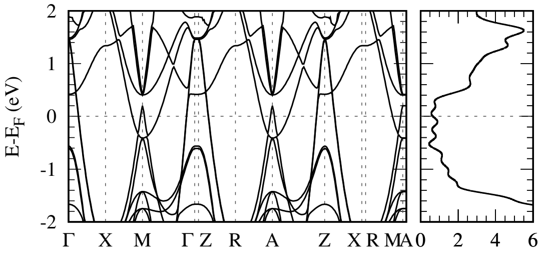

(o) TiC | LiH | TiC at $\delta_{\max} = 0$ h$^+$

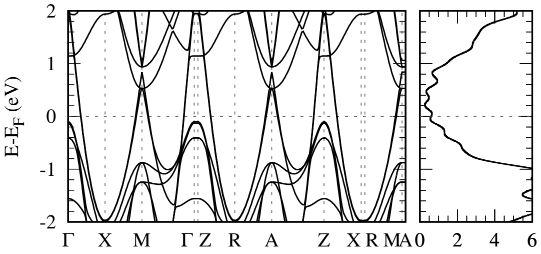

(p) TiC | LiH | TiC at $\delta_{\max} = 0.40$ h$^+$

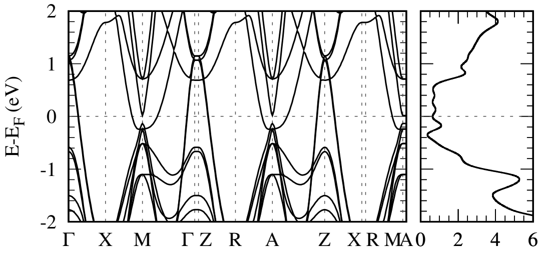

(q) ZrC | LiH | ZrC at $\delta_{\max} = 0$ h$^+$

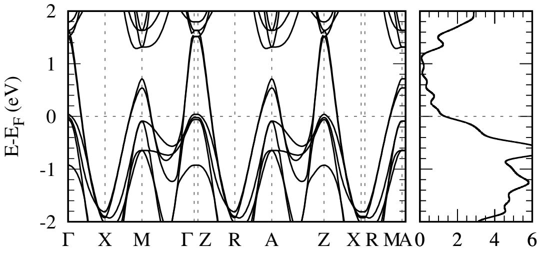

(r) ZrC | LiH | ZrC at $\delta_{\max} = 0.61$ h$^+$

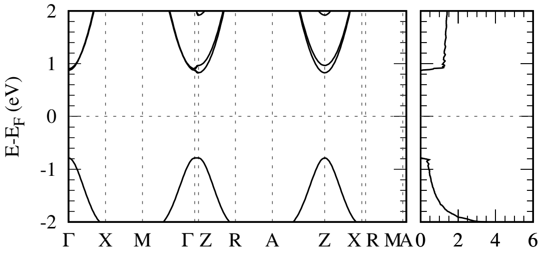

(s) $KMgF_3$ | LiH | $KMgF_3$ at $\delta_{\max} = 0$ h$^+$

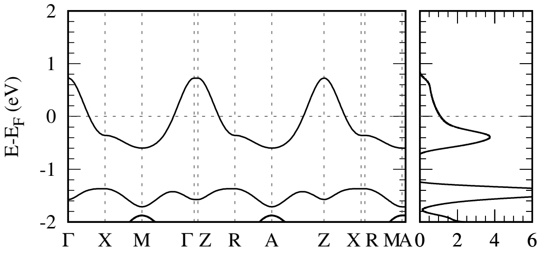

(t) $KMgF_3$ | LiH | $KMgF_3$ at $\delta_{\max} = 0.25$ h$^+$

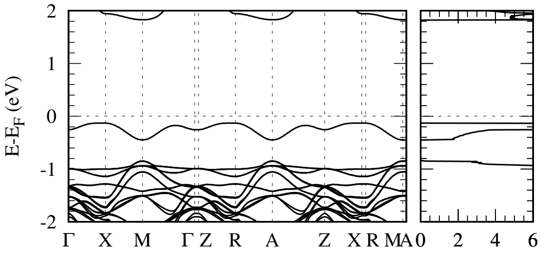

(u) $(KMgF_3)_2$ | LiH | $(KMgF_3)_2$ at $\delta_{\max} = 0$ h$^+$

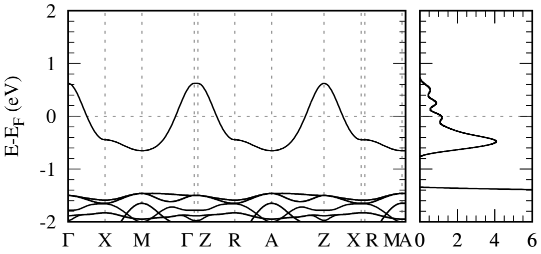

(v) $(KMgF_3)_2$ | LiH | $(KMgF_3)_2$ at $\delta_{\max} = 0.20$ h$^+$

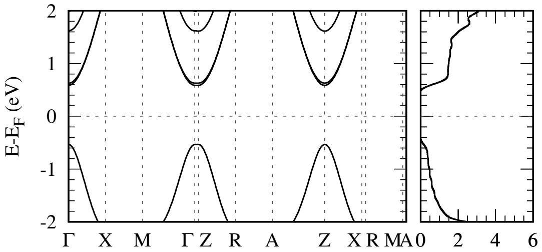

(w) $LiBaF_3$ | LiH | $LiBaF_3$ at $\delta_{\max} = 0$ h$^+$

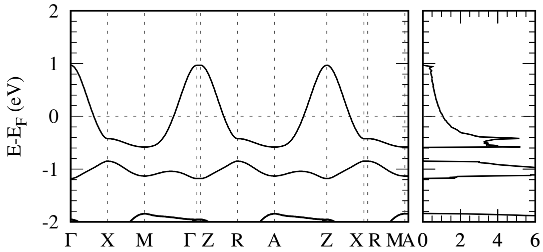

(x) $LiBaF_3$ | LiH | $LiBaF_3$ at $\delta_{\max} = 0.31$ h$^+$

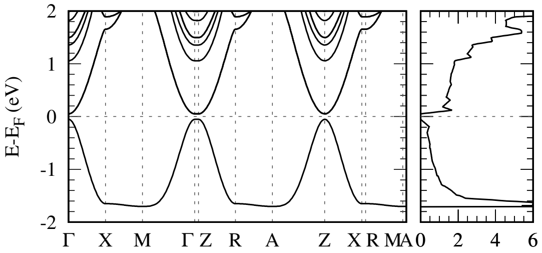

(y) $(LiBaF_3)_2$ | LiH | $(LiBaF_3)_2$ at $\delta_{\max} = 0$ h$^+$

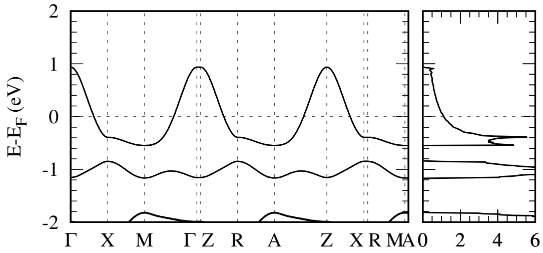

(z) $(LiBaF_3)_2$ | LiH | $(LiBaF_3)_2$ at $\delta_{\max} = 0.31$ h$^+$

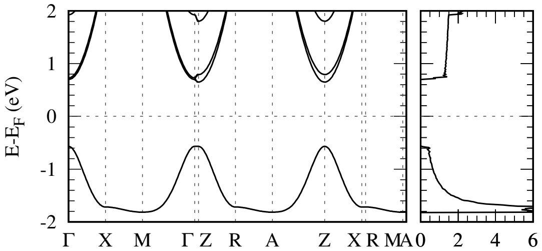

(aa) $RbMgF_3$ | LiH | $RbMgF_3$ at $\delta_{\max} = 0$ h$^+$

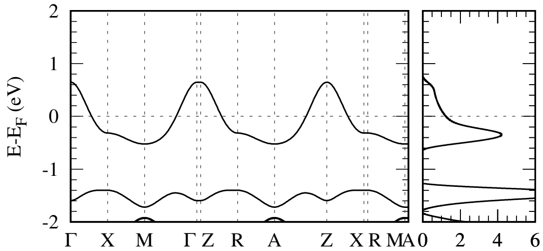

(bb) $RbMgF_3$ | LiH | $RbMgF_3$ at $\delta_{\max} = 0.25$ h$^+$

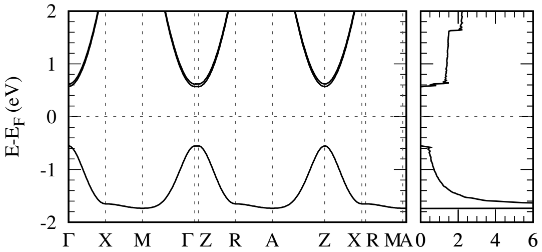

(cc) $(RbMgF_3)_2$ | LiH | $(RbMgF_3)_2$ at $\delta_{\max} = 0$ h$^+$

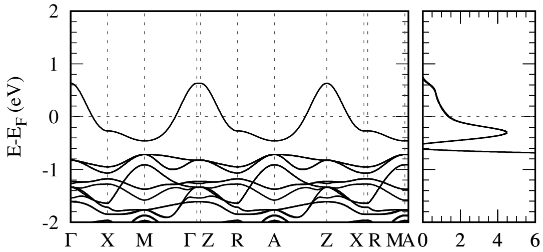

(dd) $(RbMgF_3)_2$ | LiH | $(RbMgF_3)_2$ at $\delta_{\max} = 0.27$ h$^+$

FIG. S1: Left panel: Band structure and DOS for all 15 systems at $\delta = 0$. Right panel: Band structure and DOS for all systems considered at $\delta_{\max}$.

S3. Vibrational and superconducting properties

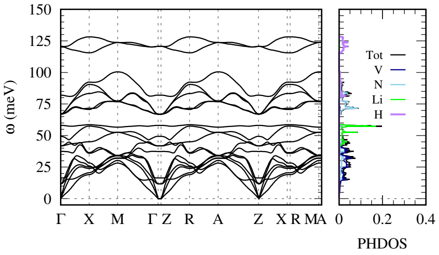

(a) VN | LiH | VN at $\delta_{\mathrm{max}} = 0$ h$^+$

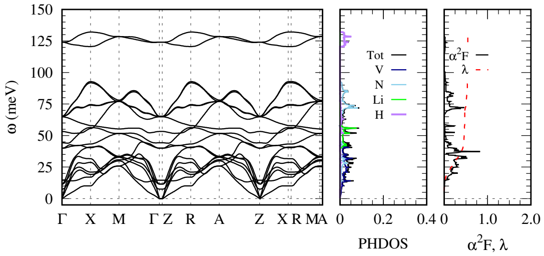

(b) VN | LiH | VN at $\delta_{\mathrm{max}} = 0.32$ h$^+$

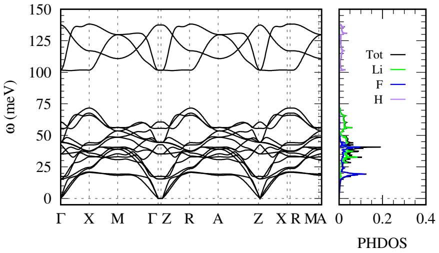

(c) LiF | LiH | LiF at $\delta_{\mathrm{max}} = 0$ h$^+$

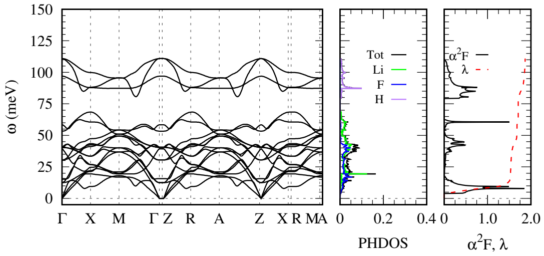

(d) LiF | LiH | LiF at $\delta_{\mathrm{max}} = 0.31$ h$^+$

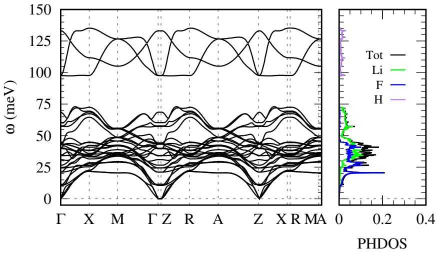

(e) $(LiF)_2$ | LiH | $(LiF)_2$ at $\delta_{\mathrm{max}} = 0$ h$^+$

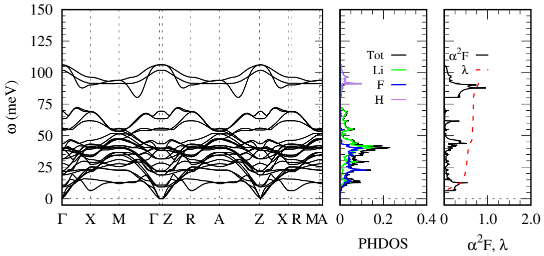

(f) $(LiF)_2$ | LiH | $(LiF)_2$ at $\delta_{\mathrm{max}} = 0.29$ h$^+$

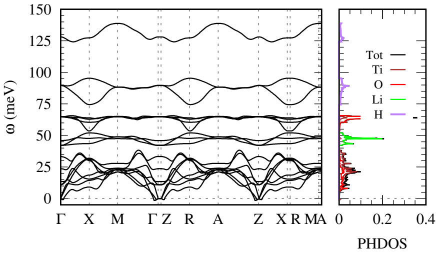

(g) TiO | LiH | TiO at $\delta_{\mathrm{max}} = 0$ h$^+$

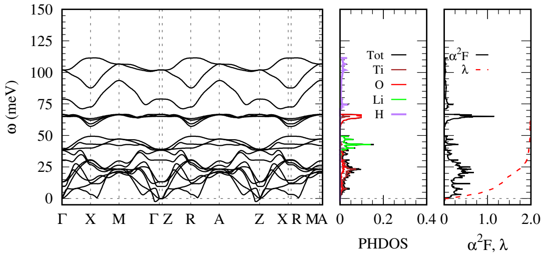

(h) TiO | LiH | TiO at $\delta_{\mathrm{max}} = 0.47$ h$^+$

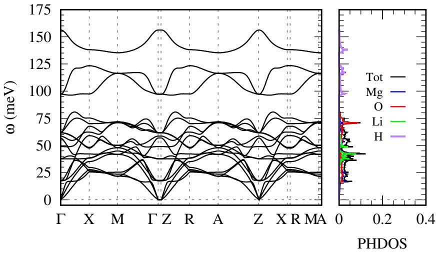

(i) MgO | LiH | MgO at $\delta_{\mathrm{max}} = 0$ h$^+$

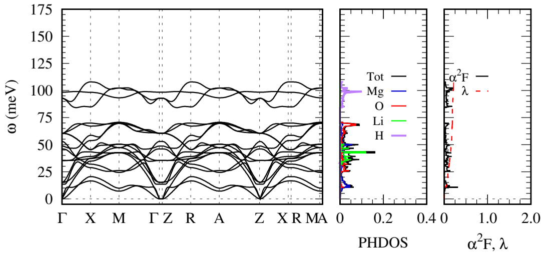

(j) MgO | LiH | MgO at $\delta_{\mathrm{max}} = 0.44$ h$^+$

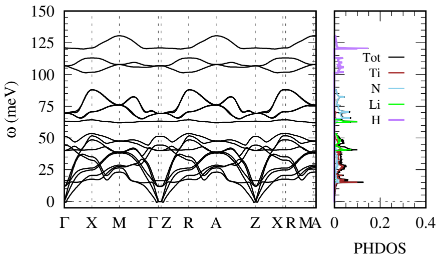

(k) TiN | LiH | TiN at $\delta_{\max} = 0$ h^{+}

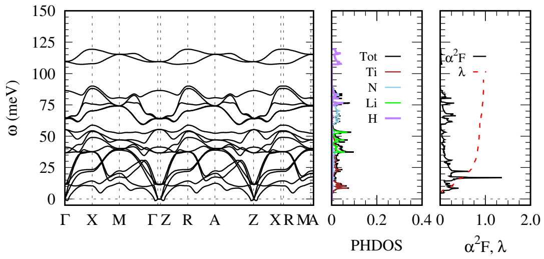

(l) TiN | LiH | TiN at $\delta_{\max} = 0.59$ h^{+}

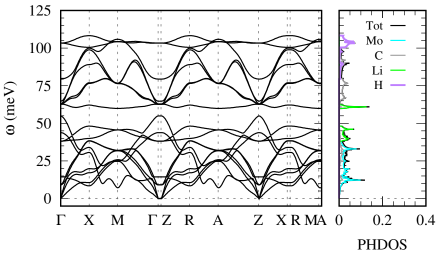

(m) MoC | LiH | MoC at $\delta_{\max} = 0$ h^{+}

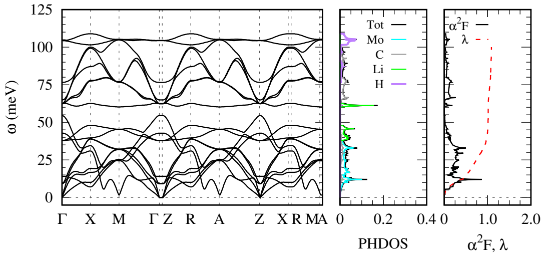

(n) MoC | LiH | MoC at $\delta_{\max} = 0.15$ h^{+}

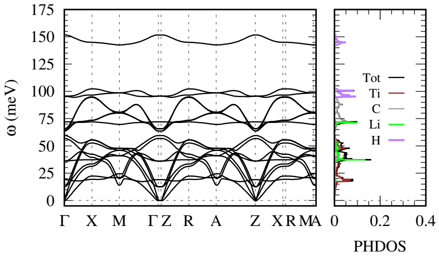

(o) TiC | LiH | TiC at $\delta_{\max} = 0$ h^{+}

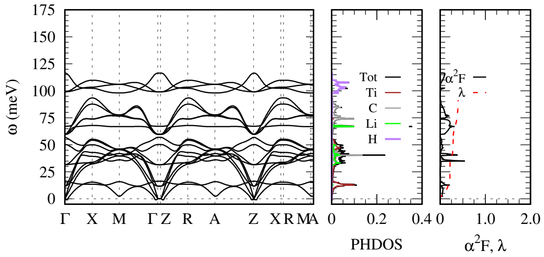

(p) TiC | LiH | TiC at $\delta_{\max} = 0.40$ h^{+}

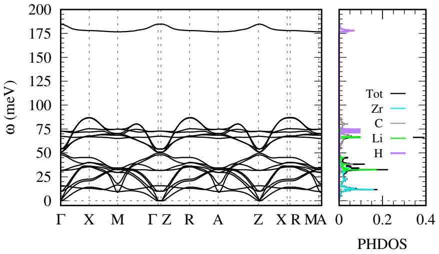

(q) ZrC | LiH | ZrC at $\delta_{\max} = 0$ h^{+}

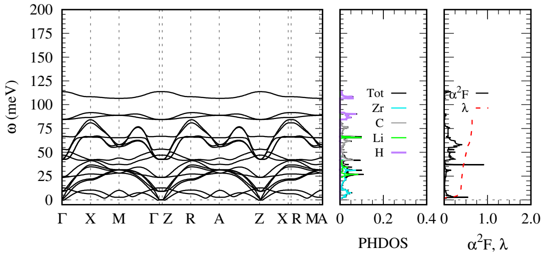

(r) ZrC | LiH | ZrC at $\delta_{\max} = 0.61$ h^{+}

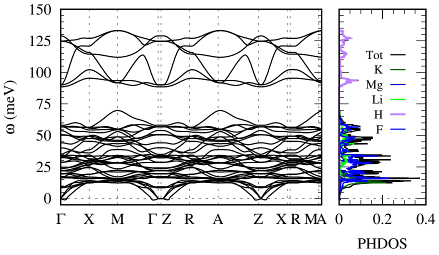

(s) $KMgF_3$ | LiH | $KMgF_3$ at $\delta_{\max} = 0$ h^{+}

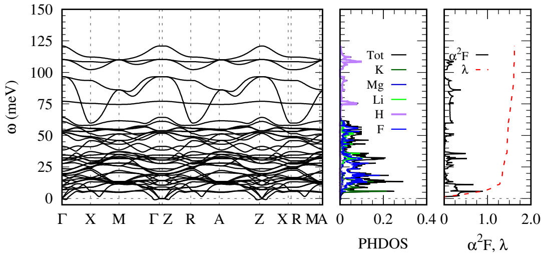

(t) $KMgF_3$ | LiH | $KMgF_3$ at $\delta_{\max} = 0.25$ h^{+}

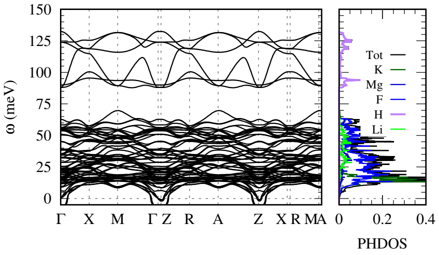

(u) $(KMgF_3)_2$ | LiH | $(KMgF_3)_2$ at $\delta_{\max} = 0$ h^{+}

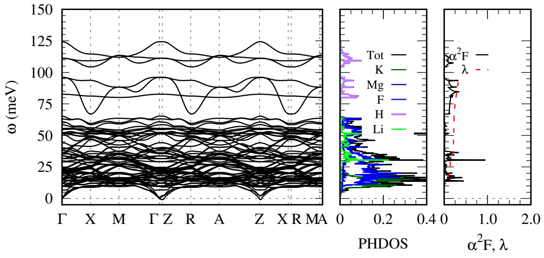

(v) $(KMgF_3)_2$ | LiH | $(KMgF_3)_2$ at $\delta_{\max} = 0.20$ h^{+}

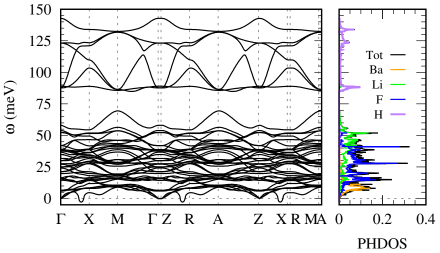

(w) $LiBaF_3$ | LiH | $LiBaF_3$ at $\delta_{max} = 0$ h^+

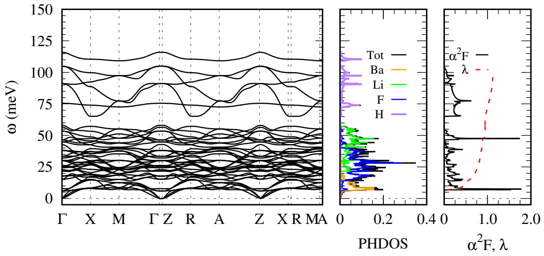

(x) $LiBaF_3$ | LiH | $LiBaF_3$ at $\delta_{max} = 0.31$ h^+

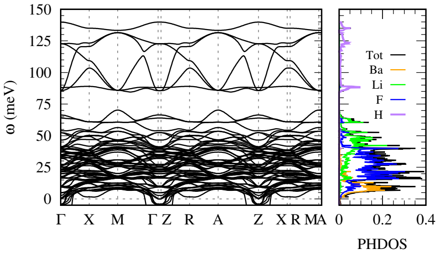

(y) $(LiBaF_3)_2$ | LiH | $(LiBaF_3)_2$ at $\delta_{max} = 0$ h^+

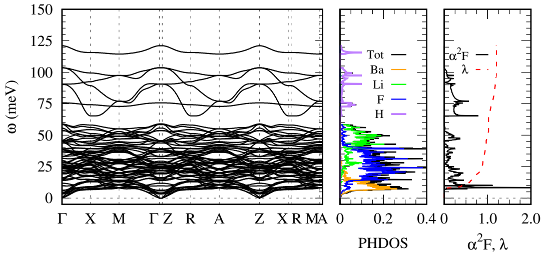

(z) $(LiBaF_3)_2$ | LiH | $(LiBaF_3)_2$ at $\delta_{max} = 0.31$ h^+

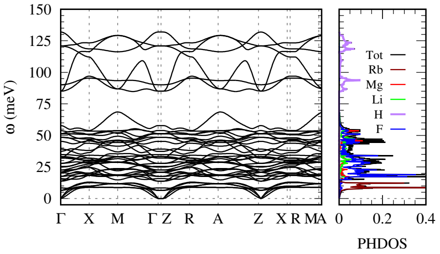

(aa) $RbMgF_3$ | LiH | $RbMgF_3$ at $\delta_{max} = 0$ h^+

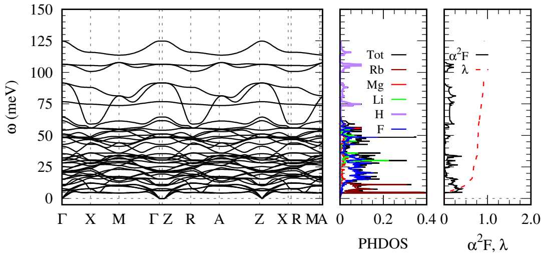

(bb) $RbMgF_3$ | LiH | $RbMgF_3$ at $\delta_{max} = 0.25$ h^+

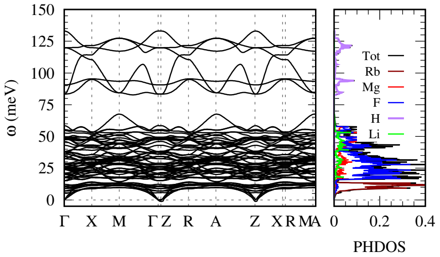

(cc) $(RbMgF_3)_2$ | LiH | $(RbMgF_3)_2$ at $\delta_{max} = 0$ h^+

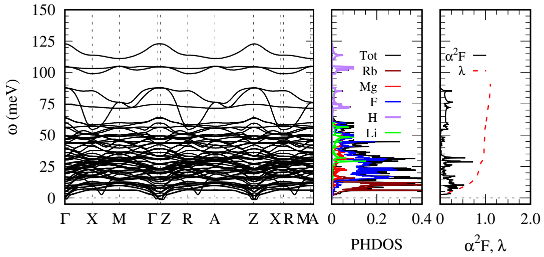

(dd) $(RbMgF_3)_2$ | LiH | $(RbMgF_3)_2$ at $\delta_{max} = 0.27$ h^+

FIG. S2: Left panel: Phonon dispersion and phonon DOS for all 15 systems at $\delta = 0$. Right panel: Phonon dispersion, phonon DOS, Eliashberg spectral function α^2F, and integrated electron-phonon coupling strength λ for all systems considered at δ_{max}.

S4. Dependence with doping for $(RbMgF_3)_2$ | LiH | $(RbMgF_3)_2$

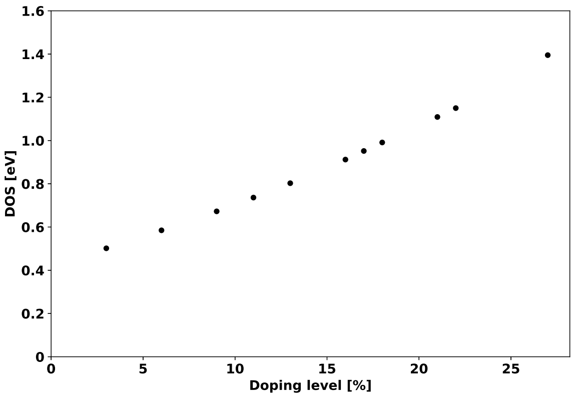

FIG. S3: Electronic density of states at Fermi level as a function of doping for $(RbMgF_3)_2$ | LiH | $(RbMgF_3)_2$.

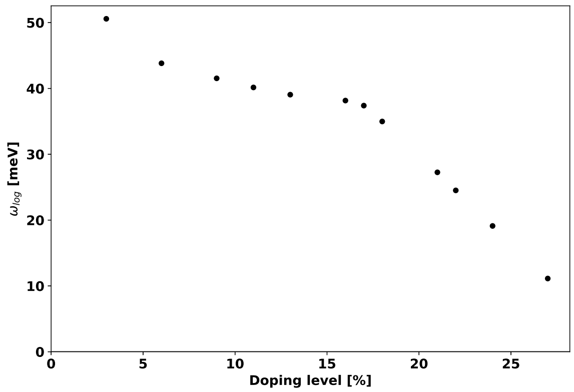

FIG. S4: Logarithmic average phonon frequency as a function of doping for $(RbMgF_3)_2$ | LiH | $(RbMgF_3)_2$.

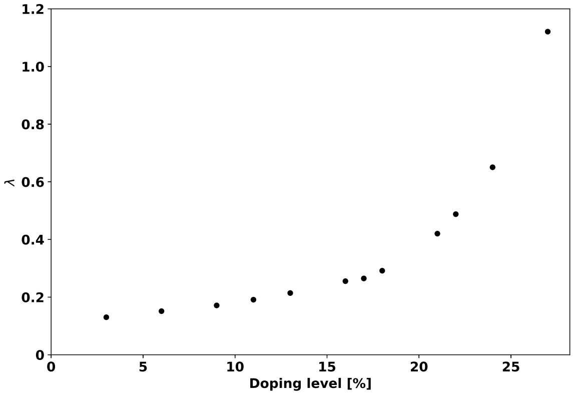

FIG. S5: Electron-phonon coupling strength as a function of doping for $(RbMgF_3)_2$ | LiH | $(RbMgF_3)_2$.

S5. Crystal properties at zero and max doping

VN | LiH | VN - 0%

```
# CIF file created by FINDSYM, version 7.1.3
data_findsym-output
_audit_creation_method FINDSYM

_cell_length_a 2.7611000000
_cell_length_b 2.7611000000
_cell_length_c 24.7200000000
_cell_angle_alpha 90.0000000000
_cell_angle_beta 90.0000000000
_cell_angle_gamma 90.0000000000
_cell_volume 188.4572017512

_symmetry_space_group_name_H-M "P 4/m 2/m 2/m"
_symmetry_Int_Tables_number 123
_space_group.reference_setting '123:-P 4 2'
_space_group.transform_Pp_abc a,b,c;0,0,0

loop_
_space_group_symop_id
_space_group_symop_operation_xyz
1 x,y,z
2 x,-y,-z
3 -x,y,-z
4 -x,-y,z
5 -y,-x,-z
6 -y,x,z
7 y,-x,z
8 y,x,-z
9 -x,-y,-z
10 -x,y,z
11 x,-y,z
12 x,y,-z
13 y,x,z
14 y,-x,-z
15 -y,x,-z
16 -y,-x,z

loop_
_atom_type_symbol
V
N
Li
H
loop_
_atom_site_label
_atom_site_type_symbol
_atom_site_symmetry_multiplicity
_atom_site_Wyckoff_symbol
_atom_site_fract_x
_atom_site_fract_y
_atom_site_fract_z
_atom_site_occupancy
_atom_site_fract_symmform
```

V1 V 2 h 0.5000000000 0.5000000000 0.4197300000 1.0000000000 0,0,Dz
N1 N 2 g 0.0000000000 0.0000000000 0.4121100000 1.0000000000 0,0,Dz
Li1 Li 1 b 0.0000000000 0.0000000000 0.5000000000 1.0000000000 0,0,0
H1 H 1 d 0.5000000000 0.5000000000 0.5000000000 1.0000000000 0,0,0

end of cif

VN | LiH | VN - 32%

CIF file created by FINDSYM, version 7.1.3

data_findsym-output
_audit_creation_method FINDSYM

_cell_length_a 2.7611000000
_cell_length_b 2.7611000000
_cell_length_c 24.7200000000
_cell_angle_alpha 90.0000000000
_cell_angle_beta 90.0000000000
_cell_angle_gamma 90.0000000000
_cell_volume 188.4572017512

_symmetry_space_group_name_H-M "P 4/m 2/m 2/m"
_symmetry_Int_Tables_number 123
_space_group.reference_setting '123:-P 4 2'
_space_group.transform_Pp_abc a,b,c;0,0,0

loop_
_space_group_symop_id
_space_group_symop_operation_xyz
1 x,y,z
2 x,-y,-z
3 -x,y,-z
4 -x,-y,z
5 -y,-x,-z
6 -y,x,z
7 y,-x,z
8 y,x,-z
9 -x,-y,-z
10 -x,y,z
11 x,-y,z
12 x,y,-z
13 y,x,z
14 y,-x,-z
15 -y,x,-z
16 -y,-x,z

loop_
_atom_type_symbol
V
N
Li
H

loop_
_atom_site_label
_atom_site_type_symbol

_atom_site_symmetry_multiplicity
_atom_site_Wyckoff_symbol
_atom_site_fract_x
_atom_site_fract_y
_atom_site_fract_z
_atom_site_occupancy
_atom_site_fract_symmform
V1 V 2 h 0.5000000000 0.5000000000 0.4175700000 1.0000000000 0,0,Dz
N1 N 2 g 0.0000000000 0.0000000000 0.4122900000 1.0000000000 0,0,Dz
Li1 Li 1 b 0.0000000000 0.0000000000 0.5000000000 1.0000000000 0,0,0
H1 H 1 d 0.5000000000 0.5000000000 0.5000000000 1.0000000000 0,0,0

end of cif

LiF | LiH | LiF - 0%

CIF file created by FINDSYM, version 7.1.3
data_findsym-output
_audit_creation_method FINDSYM
_cell_length_a 2.7729000000
_cell_length_b 2.7729000000
_cell_length_c 18.4964000000
_cell_angle_alpha 90.0000000000
_cell_angle_beta 90.0000000000
_cell_angle_gamma 90.0000000000
_cell_volume 142.2183462771
_symmetry_space_group_name_H-M "P 4/m 2/m 2/m"
_symmetry_Int_Tables_number 123
_space_group.reference_setting '123:-P 4 2'
_space_group.transform_Pp_abc a,b,c;0,0,0
loop_
_space_group_symop_id
_space_group_symop_operation_xyz
1 x,y,z
2 x,-y,-z
3 -x,y,-z
4 -x,-y,z
5 -y,-x,-z
6 -y,x,z
7 y,-x,z
8 y,x,-z
9 -x,-y,-z
10 -x,y,z
11 x,-y,z
12 x,y,-z
13 y,x,z
14 y,-x,-z
15 -y,x,-z
16 -y,-x,z
loop_
_atom_site_label
_atom_site_type_symbol
_atom_site_symmetry_multiplicity
_atom_site_Wyckoff_symbol
_atom_site_fract_x
_atom_site_fract_y
_atom_site_fract_z

_atom_site_occupancy
_atom_site_fract_symmform
Li1 Li 2 g 0.00000 0.00000 0.39077 1.00000 0,0,Dz
Li2 Li 1 d 0.50000 0.50000 0.50000 1.00000 0,0,0
F1 F 2 h 0.50000 0.50000 0.38766 1.00000 0,0,Dz
H1 H 1 b 0.00000 0.00000 0.50000 1.00000 0,0,0
end of cif

LiF | LiH | LiF - 31%

CIF file created by FINDSYM, version 7.1.3
data_findsym-output
_audit_creation_method FINDSYM

_cell_length_a 2.7762000000
_cell_length_b 2.7762000000
_cell_length_c 18.4964000000
_cell_angle_alpha 90.0000000000
_cell_angle_beta 90.0000000000
_cell_angle_gamma 90.0000000000
_cell_volume 142.5570529088

_symmetry_space_group_name_H-M "P 4/m 2/m 2/m" _symmetry_Int_Tables_number 123 _space_group.reference_setting '123:-P 4 2' _space_group.transform_Pp_abc a,b,c;0,0,0
loop_
_space_group_symop_id _space_group_symop_operation_xyz 1 x,y,z
2 x,-y,-z
3 -x,y,-z
4 -x,-y,z
5 -y,-x,-z
6 -y,x,z
7 y,-x,z
8 y,x,-z
9 -x,-y,-z
10 -x,y,z
11 x,-y,z
12 x,y,-z
13 y,x,z
14 y,-x,-z
15 -y,x,-z
16 -y,-x,z

loop_
_atom_type_symbol
Li
F
H

loop_
_atom_site_label
_atom_site_type_symbol
_atom_site_symmetry_multiplicity
_atom_site_Wyckoff_symbol
_atom_site_fract_x
_atom_site_fract_y
_atom_site_fract_z
_atom_site_occupancy
_atom_site_fract_symmform

Li1 Li 2 g 0.0000000000 0.0000000000 0.3754300000 1.0000000000 0,0,Dz
Li2 Li 1 d 0.5000000000 0.5000000000 0.5000000000 1.0000000000 0,0,0
F1 F 2 h 0.5000000000 0.5000000000 0.3880800000 1.0000000000 0,0,Dz
H1 H 1 b 0.0000000000 0.0000000000 0.5000000000 1.0000000000 0,0,0

end of cif

$(LiF)_2$ | LiH | $(LiF)_2$ - 0%

CIF file created by FINDSYM, version 7.1.3
data_findsym-output
_audit_creation_method FINDSYM
_cell_length_a 2.8102000000
_cell_length_b 2.8102000000
_cell_length_c 21.9721000000
_cell_angle_alpha 90.0000000000
_cell_angle_beta 90.0000000000
_cell_angle_gamma 90.0000000000
_cell_volume 173.5185963293
_symmetry_space_group_name_H-M "P 4/m 2/m 2/m"
_symmetry_Int_Tables_number 123
_space_group.reference_setting '123:-P 4 2'
_space_group.transform_Pp_abc a,b,c;0,0,0

loop_
_space_group_symop_id _space_group_symop_operation_xyz 1 x,y,z
2 x,-y,-z
3 -x,y,-z
4 -x,-y,z
5 -y,-x,-z
6 -y,x,z
7 y,-x,z
8 y,x,-z
9 -x,-y,-z
10 -x,y,z
11 x,-y,z
12 x,y,-z
13 y,x,z
14 y,-x,-z
15 -y,x,-z
16 -y,-x,z

loop_
_atom_type_symbol
Li
F
H

loop_
_atom_site_label
_atom_site_type_symbol
_atom_site_symmetry_multiplicity
_atom_site_Wyckoff_symbol
_atom_site_fract_x
_atom_site_fract_y
_atom_site_fract_z
_atom_site_occupancy
_atom_site_fract_symmform

Li1 Li 2 h 0.5000000000 0.5000000000 0.6855000000 1.0000000000 0,0,Dz
Li2 Li 2 g 0.0000000000 0.0000000000 0.5938900000 1.0000000000 0,0,Dz
Li3 Li 1 d 0.5000000000 0.5000000000 0.5000000000 1.0000000000 0,0,0
F1 F 2 g 0.0000000000 0.0000000000 0.6885100000 1.0000000000 0,0,Dz
F2 F 2 h 0.5000000000 0.5000000000 0.5934000000 1.0000000000 0,0,Dz
H1 H 1 b 0.0000000000 0.0000000000 0.5000000000 1.0000000000 0,0,0
end of cif

$(LiF)_2$ | LiH | $(LiF)_2$ - 29%

CIF file created by FINDSYM, version 7.1.3 data_findsym-output
_audit_creation_method FINDSYM
_cell_length_a 2.8102000000
_cell_length_b 2.8102000000
_cell_length_c 21.9721000000
_cell_angle_alpha 90.0000000000
_cell_angle_beta 90.0000000000
_cell_angle_gamma 90.0000000000
_cell_volume 173.5185963293

_symmetry_space_group_name_H-M "P 4/m 2/m 2/m"
_symmetry_Int_Tables_number 123
_space_group.reference_setting '123:-P 4 2'
_space_group.transform_Pp_abc a,b,c;0,0,0

loop_
_space_group_symop_id
_space_group_symop_operation_xyz
1 x,y,z
2 x,-y,-z
3 -x,y,-z
4 -x,-y,z
5 -y,-x,-z
6 -y,x,z
7 y,-x,z
8 y,x,-z
9 -x,-y,-z
10 -x,y,z
11 x,-y,z
12 x,y,-z
13 y,x,z
14 y,-x,-z
15 -y,x,-z
16 -y,-x,z

loop_
_atom_type_symbol
Li
F
H

loop_
_atom_site_label
_atom_site_type_symbol
_atom_site_symmetry_multiplicity
_atom_site_Wyckoff_symbol
_atom_site_fract_x
_atom_site_fract_y

_atom_site_fract_z
_atom_site_occupancy
_atom_site_fract_symmform
Li1 Li 2 h 0.5000000000 0.5000000000 0.6855000000 1.0000000000 0,0,Dz
Li2 Li 2 g 0.0000000000 0.0000000000 0.5938900000 1.0000000000 0,0,Dz
Li3 Li 1 d 0.5000000000 0.5000000000 0.5000000000 1.0000000000 0,0,0
F1 F 2 g 0.0000000000 0.0000000000 0.6885100000 1.0000000000 0,0,Dz
F2 F 2 h 0.5000000000 0.5000000000 0.5934000000 1.0000000000 0,0,Dz
H1 H 1 b 0.0000000000 0.0000000000 0.5000000000 1.0000000000 0,0,0

end of cif

TiO | LiH | TiO - 0%

CIF file created by FINDSYM, version 7.1.3
data_findsym-output
_audit_creation_method FINDSYM
_cell_length_a 2.8744000000
_cell_length_b 2.8744000000
_cell_length_c 24.7200000000
_cell_angle_alpha 90.0000000000
_cell_angle_beta 90.0000000000
_cell_angle_gamma 90.0000000000
_cell_volume 204.2409748992

_symmetry_space_group_name_H-M "P 4/m 2/m 2/m"
_symmetry_Int_Tables_number 123
_space_group.reference_setting '123:-P 4 2'
_space_group.transform_Pp_abc a,b,c;0,0,0

loop_
_space_group_symop_id
_space_group_symop_operation_xyz
1 x,y,z
2 x,-y,-z
3 -x,y,-z
4 -x,-y,z
5 -y,-x,-z
6 -y,x,z
7 y,-x,z
8 y,x,-z
9 -x,-y,-z
10 -x,y,z
11 x,-y,z
12 x,y,-z
13 y,x,z
14 y,-x,-z
15 -y,x,-z
16 -y,-x,z

loop_
_atom_type_symbol
Ti
O
Li
H

loop_

_atom_site_label
_atom_site_type_symbol
_atom_site_symmetry_multiplicity
_atom_site_Wyckoff_symbol
_atom_site_fract_x
_atom_site_fract_y
_atom_site_fract_z
_atom_site_occupancy
_atom_site_fract_symmform
Ti1 Ti 2 h 0.5000000000 0.5000000000 0.4191600000 1.0000000000 0,0,Dz
O1 O 2 g 0.0000000000 0.0000000000 0.4115000000 1.0000000000 0,0,Dz
Li1 Li 1 b 0.0000000000 0.0000000000 0.5000000000 1.0000000000 0,0,0
H1 H 1 d 0.5000000000 0.5000000000 0.5000000000 1.0000000000 0,0,0

end of cif

TiO | LiH | TiO - 47%

CIF file created by FINDSYM, version 7.1.3

data_findsym-output
_audit_creation_method FINDSYM

_cell_length_a 2.8744000000
_cell_length_b 2.8744000000
_cell_length_c 24.7200000000
_cell_angle_alpha 90.0000000000
_cell_angle_beta 90.0000000000
_cell_angle_gamma 90.0000000000
_cell_volume 204.2409748992

_symmetry_space_group_name_H-M "P 4/m 2/m 2/m"
_symmetry_Int_Tables_number 123
_space_group.reference_setting '123:-P 4 2'
_space_group.transform_Pp_abc a,b,c;0,0,0

loop_
_space_group_symop_id
_space_group_symop_operation_xyz
1 x,y,z
2 x,-y,-z
3 -x,y,-z
4 -x,-y,z
5 -y,-x,-z
6 -y,x,z
7 y,-x,z
8 y,x,-z
9 -x,-y,-z
10 -x,y,z
11 x,-y,z
12 x,y,-z
13 y,x,z
14 y,-x,-z
15 -y,x,-z
16 -y,-x,z

loop_

_atom_type_symbol
Ti
O
Li
H

loop_
_atom_site_label
_atom_site_type_symbol
_atom_site_symmetry_multiplicity
_atom_site_Wyckoff_symbol
_atom_site_fract_x
_atom_site_fract_y
_atom_site_fract_z
_atom_site_occupancy
_atom_site_fract_symmform
Ti1 Ti 2 h 0.5000000000 0.5000000000 0.4139900000 1.0000000000 0,0,Dz
O1 O 2 g 0.0000000000 0.0000000000 0.4100300000 1.0000000000 0,0,Dz
Li1 Li 1 b 0.0000000000 0.0000000000 0.5000000000 1.0000000000 0,0,0
H1 H 1 d 0.5000000000 0.5000000000 0.5000000000 1.0000000000 0,0,0
end of cif

MgO | LiH | MgO - 0%

CIF file created by FINDSYM, version 7.1.3
data_findsym-output
_audit_creation_method FINDSYM

_cell_length_a 2.8778000000
_cell_length_b 2.8778000000
_cell_length_c 24.7200000000
_cell_angle_alpha 90.0000000000
_cell_angle_beta 90.0000000000
_cell_angle_gamma 90.0000000000
_cell_volume 204.7244358048

_symmetry_space_group_name_H-M ”P 4/m 2/m 2/m”
_symmetry_Int_Tables_number 123
_space_group.reference_setting ’123:-P 4 2’
_space_group.transform_Pp_abc a,b,c;0,0,0

loop_
_space_group_symop_id
_space_group_symop_operation_xyz
1 x,y,z
2 x,-y,-z
3 -x,y,-z
4 -x,-y,z
5 -y,-x,-z
6 -y,x,z
7 y,-x,z
8 y,x,-z
9 -x,-y,-z
10 -x,y,z
11 x,-y,z
12 x,y,-z
13 y,x,z

14 y,-x,-z
15 -y,x,-z
16 -y,-x,z

loop_
_atom_type_symbol
Mg
O
Li
H

loop_
_atom_site_label
_atom_site_type_symbol
_atom_site_symmetry_multiplicity
_atom_site_Wyckoff_symbol
_atom_site_fract_x
_atom_site_fract_y
_atom_site_fract_z
_atom_site_occupancy
_atom_site_fract_symmform
Mg1 Mg 2 h 0.5000000000 0.5000000000 0.4159000000 1.0000000000 0,0,Dz
O1 O 2 g 0.0000000000 0.0000000000 0.4149300000 1.0000000000 0,0,Dz
Li1 Li 1 b 0.0000000000 0.0000000000 0.5000000000 1.0000000000 0,0,0
H1 H 1 d 0.5000000000 0.5000000000 0.5000000000 1.0000000000 0,0,0

end of cif

MgO | LiH | MgO - 44%

CIF file created by FINDSYM, version 7.1.3

data_findsym-output
_audit_creation_method FINDSYM

_cell_length_a 2.8778000000
_cell_length_b 2.8778000000
_cell_length_c 24.7200000000
_cell_angle_alpha 90.0000000000
_cell_angle_beta 90.0000000000
_cell_angle_gamma 90.0000000000
_cell_volume 204.7244358048

_symmetry_space_group_name_H-M "P 4/m 2/m 2/m"
_symmetry_Int_Tables_number 123
_space_group.reference_setting '123:-P 4 2'
_space_group.transform_Pp_abc a,b,c;0,0,0

loop_
_space_group_symop_id
_space_group_symop_operation_xyz
1 x,y,z
2 x,-y,-z
3 -x,y,-z
4 -x,-y,z
5 -y,-x,-z
6 -y,x,z

7 y,-x,z
8 y,x,-z
9 -x,-y,-z
10 -x,y,z
11 x,-y,z
12 x,y,-z
13 y,x,z
14 y,-x,-z
15 -y,x,-z
16 -y,-x,z

loop_
_atom_type_symbol
Mg
O
Li
H

loop_
_atom_site_label
_atom_site_type_symbol
_atom_site_symmetry_multiplicity
_atom_site_Wyckoff_symbol
_atom_site_fract_x
_atom_site_fract_y
_atom_site_fract_z
_atom_site_occupancy
_atom_site_fract_symmform
Mg1 Mg 2 h 0.5000000000 0.5000000000 0.4091700000 1.0000000000 0,0,Dz
O1 O 2 g 0.0000000000 0.0000000000 0.4159050000 1.0000000000 0,0,Dz
Li1 Li 1 b 0.0000000000 0.0000000000 0.5000000000 1.0000000000 0,0,0
H1 H 1 d 0.5000000000 0.5000000000 0.5000000000 1.0000000000 0,0,0

end of cif

TiN | LiH | TiN - 0%

CIF file created by FINDSYM, version 7.1.3
data_findsym-output
_audit_creation_method FINDSYM
_cell_length_a 2.8776000000
_cell_length_b 2.8776000000
_cell_length_c 24.7200000000
_cell_angle_alpha 90.0000000000
_cell_angle_beta 90.0000000000
_cell_angle_gamma 90.0000000000
_cell_volume 204.6959811072
_symmetry_space_group_name_H-M "P 4/m 2/m 2/m"
_symmetry_Int_Tables_number 123
_space_group.reference_setting '123:-P 4 2'
_space_group.transform_Pp_abc a,b,c;0,0,0
loop_
_space_group_symop_id
_space_group_symop_operation_xyz
1 x,y,z
2 x,-y,-z
3 -x,y,-z

4 -x,-y,z
5 -y,-x,-z
6 -y,x,z
7 y,-x,z
8 y,x,-z
9 -x,-y,-z
10 -x,y,z
11 x,-y,z
12 x,y,-z
13 y,x,z
14 y,-x,-z
15 -y,x,-z
16 -y,-x,z
loop_
_atom_type_symbol
Ti
N
Li
H
loop_
_atom_site_label
_atom_site_type_symbol
_atom_site_symmetry_multiplicity
_atom_site_Wyckoff_symbol
_atom_site_fract_x
_atom_site_fract_y
_atom_site_fract_z
_atom_site_occupancy
_atom_site_fract_symmform
Ti1 Ti 2 h 0.5000000000 0.5000000000 0.4175700000 1.0000000000 0,0,Dz
N1 N 2 g 0.0000000000 0.0000000000 0.4150900000 1.0000000000 0,0,Dz
Li1 Li 1 b 0.0000000000 0.0000000000 0.5000000000 1.0000000000 0,0,0
H1 H 1 d 0.5000000000 0.5000000000 0.5000000000 1.0000000000 0,0,0
end of cif

TiN | LiH | TiN - 59%

CIF file created by FINDSYM, version 7.1.3
data_findsym-output
_audit_creation_method FINDSYM
_cell_length_a 2.8776000000
_cell_length_b 2.8776000000
_cell_length_c 24.7200000000
_cell_angle_alpha 90.0000000000
_cell_angle_beta 90.0000000000
_cell_angle_gamma 90.0000000000
_cell_volume 204.6959811072
_symmetry_space_group_name_H-M "P 4/m 2/m 2/m"
_symmetry_Int_Tables_number 123
_space_group.reference_setting '123:-P 4 2'
_space_group.transform_Pp_abc a,b,c;0,0,0
loop_
_space_group_symop_id
_space_group_symop_operation_xyz
1 x,y,z
2 x,-y,-z
3 -x,y,-z

4 -x,-y,z
5 -y,-x,-z
6 -y,x,z
7 y,-x,z
8 y,x,-z
9 -x,-y,-z
10 -x,y,z
11 x,-y,z
12 x,y,-z
13 y,x,z
14 y,-x,-z
15 -y,x,-z
16 -y,-x,z
loop_
_atom_type_symbol
Ti
N
Li
H
loop_
_atom_site_label
_atom_site_type_symbol
_atom_site_symmetry_multiplicity
_atom_site_Wyckoff_symbol
_atom_site_fract_x
_atom_site_fract_y
_atom_site_fract_z
_atom_site_occupancy
_atom_site_fract_symmform
Ti1 Ti 2 h 0.5000000000 0.5000000000 0.4116600000 1.0000000000 0,0,Dz
N1 N 2 g 0.0000000000 0.0000000000 0.4125500000 1.0000000000 0,0,Dz
Li1 Li 1 b 0.0000000000 0.0000000000 0.5000000000 1.0000000000 0,0,0
H1 H 1 d 0.5000000000 0.5000000000 0.5000000000 1.0000000000 0,0,0
end of cif

MoC | LiH | MoC - 0%

CIF file created by FINDSYM, version 7.1.3

data_findsym-output
_audit_creation_method FINDSYM

_cell_length_a 2.9372000000
_cell_length_b 2.9372000000
_cell_length_c 24.7200000000
_cell_angle_alpha 90.0000000000
_cell_angle_beta 90.0000000000
_cell_angle_gamma 90.0000000000
_cell_volume 213.2629957248

_symmetry_space_group_name_H-M ”P 4/m 2/m 2/m”
_symmetry_Int_Tables_number 123
_space_group.reference_setting ’123:-P 4 2’
_space_group.transform_Pp_abc a,b,c;0,0,0

loop_
_space_group_symop_id

_space_group_symop_operation_xyz
1 x,y,z
2 x,-y,-z
3 -x,y,-z
4 -x,-y,z
5 -y,-x,-z
6 -y,x,z
7 y,-x,z
8 y,x,-z
9 -x,-y,-z
10 -x,y,z
11 x,-y,z
12 x,y,-z
13 y,x,z
14 y,-x,-z
15 -y,x,-z
16 -y,-x,z

loop_
_atom_type_symbol
Mo
C
Li
H

loop_
_atom_site_label
_atom_site_type_symbol
_atom_site_symmetry_multiplicity
_atom_site_Wyckoff_symbol
_atom_site_fract_x
_atom_site_fract_y
_atom_site_fract_z
_atom_site_occupancy
_atom_site_fract_symmform
Mo1 Mo 2 h 0.5000000000 0.5000000000 0.4188200000 1.0000000000 0,0,Dz
C1 C 2 g 0.0000000000 0.0000000000 0.4088400000 1.0000000000 0,0,Dz
Li1 Li 1 b 0.0000000000 0.0000000000 0.5000000000 1.0000000000 0,0,0
H1 H 1 d 0.5000000000 0.5000000000 0.5000000000 1.0000000000 0,0,0

end of cif

MoC | LiH | MoC - 15%

CIF file created by FINDSYM, version 7.1.3

data_findsym-output
_audit_creation_method FINDSYM

_cell_length_a 2.9372000000
_cell_length_b 2.9372000000
_cell_length_c 24.7200000000
_cell_angle_alpha 90.0000000000
_cell_angle_beta 90.0000000000
_cell_angle_gamma 90.0000000000
_cell_volume 213.2629957248

```
_symmetry_space_group_name_H-M "P 4/m 2/m 2/m"
_symmetry_Int_Tables_number 123
_space_group.reference_setting '123:-P 4 2'
_space_group.transform_Pp_abc a,b,c;0,0,0

loop_
_space_group_symop_id
_space_group_symop_operation_xyz
1 x,y,z
2 x,-y,-z
3 -x,y,-z
4 -x,-y,z
5 -y,-x,-z
6 -y,x,z
7 y,-x,z
8 y,x,-z
9 -x,-y,-z
10 -x,y,z
11 x,-y,z
12 x,y,-z
13 y,x,z
14 y,-x,-z
15 -y,x,-z
16 -y,-x,z

loop_
_atom_type_symbol
Mo
C
Li
H

loop_
_atom_site_label
_atom_site_type_symbol
_atom_site_symmetry_multiplicity
_atom_site_Wyckoff_symbol
_atom_site_fract_x
_atom_site_fract_y
_atom_site_fract_z
_atom_site_occupancy
_atom_site_fract_symmform
Mo1 Mo 2 h 0.5000000000 0.5000000000 0.4185800000 1.0000000000 0,0,Dz
C1 C 2 g 0.0000000000 0.0000000000 0.4091200000 1.0000000000 0,0,Dz
Li1 Li 1 b 0.0000000000 0.0000000000 0.5000000000 1.0000000000 0,0,0
H1 H 1 d 0.5000000000 0.5000000000 0.5000000000 1.0000000000 0,0,0

# end of cif
```

TiC | LiH | TiC - 0%

```
# CIF file created by FINDSYM, version 7.1.3

data_findsym-output
_audit_creation_method FINDSYM

_cell_length_a 2.9528000000
```

_cell_length_b 2.9528000000
_cell_length_c 24.7200000000
_cell_angle_alpha 90.0000000000
_cell_angle_beta 90.0000000000
_cell_angle_gamma 90.0000000000
_cell_volume 215.5343682048

_symmetry_space_group_name_H-M "P 4/m 2/m 2/m"
_symmetry_Int_Tables_number 123
_space_group.reference_setting '123:-P 4 2'
_space_group.transform_Pp_abc a,b,c;0,0,0

loop_
_space_group_symop_id
_space_group_symop_operation_xyz
1 x,y,z
2 x,-y,-z
3 -x,y,-z
4 -x,-y,z
5 -y,-x,-z
6 -y,x,z
7 y,-x,z
8 y,x,-z
9 -x,-y,-z
10 -x,y,z
11 x,-y,z
12 x,y,-z
13 y,x,z
14 y,-x,-z
15 -y,x,-z
16 -y,-x,z

loop_
_atom_type_symbol
Ti
C
Li
H

loop_
_atom_site_label
_atom_site_type_symbol
_atom_site_symmetry_multiplicity
_atom_site_Wyckoff_symbol
_atom_site_fract_x
_atom_site_fract_y
_atom_site_fract_z
_atom_site_occupancy
_atom_site_fract_symmform
Ti1 Ti 2 h 0.5000000000 0.5000000000 0.4182000000 1.0000000000 0,0,Dz
C1 C 2 g 0.0000000000 0.0000000000 0.4146500000 1.0000000000 0,0,Dz
Li1 Li 1 b 0.0000000000 0.0000000000 0.5000000000 1.0000000000 0,0,0
H1 H 1 d 0.5000000000 0.5000000000 0.5000000000 1.0000000000 0,0,0

end of cif

TiC | LiH | TiC - 40%

```
# CIF file created by FINDSYM, version 7.1.3

data_findsym-output
_audit_creation_method FINDSYM

_cell_length_a 2.9528000000
_cell_length_b 2.9528000000
_cell_length_c 24.7200000000
_cell_angle_alpha 90.0000000000
_cell_angle_beta 90.0000000000
_cell_angle_gamma 90.0000000000
_cell_volume 215.5343682048

_symmetry_space_group_name_H-M "P 4/m 2/m 2/m"
_symmetry_Int_Tables_number 123
_space_group.reference_setting '123:-P 4 2'
_space_group.transform_Pp_abc a,b,c;0,0,0

loop_
_space_group_symop_id
_space_group_symop_operation_xyz
1 x,y,z
2 x,-y,-z
3 -x,y,-z
4 -x,-y,z
5 -y,-x,-z
6 -y,x,z
7 y,-x,z
8 y,x,-z
9 -x,-y,-z
10 -x,y,z
11 x,-y,z
12 x,y,-z
13 y,x,z
14 y,-x,-z
15 -y,x,-z
16 -y,-x,z

loop_
_atom_type_symbol
Ti
C
Li
H

loop_
_atom_site_label
_atom_site_type_symbol
_atom_site_symmetry_multiplicity
_atom_site_Wyckoff_symbol
_atom_site_fract_x
_atom_site_fract_y
_atom_site_fract_z
_atom_site_occupancy
_atom_site_fract_symmform
Ti1 Ti 2 h 0.5000000000 0.5000000000 0.4134400000 1.0000000000 0,0,Dz
C1 C 2 g 0.0000000000 0.0000000000 0.4136700000 1.0000000000 0,0,Dz
```

```
Li1 Li 1 b 0.0000000000 0.0000000000 0.5000000000 1.0000000000 0,0,0
H1 H 1 d 0.5000000000 0.5000000000 0.5000000000 1.0000000000 0,0,0

# end of cif
```

ZrC | LiH | ZrC - 0%

```
# CIF file created by FINDSYM, version 7.1.3

data_findsym-output _audit_creation_method FINDSYM

_cell_length_a 3.2168000000
_cell_length_b 3.2168000000
_cell_length_c 24.7200000000
_cell_angle_alpha 90.0000000000
_cell_angle_beta 90.0000000000
_cell_angle_gamma 90.0000000000
_cell_volume 255.7976713728

_symmetry_space_group_name_H-M "P 4/m 2/m 2/m"
_symmetry_Int_Tables_number 123
_space_group.reference_setting '123:-P 4 2'
_space_group.transform_Pp_abc a,b,c;0,0,0

loop_
_space_group_symop_id
_space_group_symop_operation_xyz
1 x,y,z
2 x,-y,-z
3 -x,y,-z
4 -x,-y,z
5 -y,-x,-z
6 -y,x,z
7 y,-x,z
8 y,x,-z
9 -x,-y,-z
10 -x,y,z
11 x,-y,z
12 x,y,-z
13 y,x,z
14 y,-x,-z
15 -y,x,-z
16 -y,-x,z

loop_
_atom_type_symbol
Zr
C
Li
H

loop_
_atom_site_label
_atom_site_type_symbol
_atom_site_symmetry_multiplicity
_atom_site_Wyckoff_symbol
_atom_site_fract_x
```

_atom_site_fract_y
_atom_site_fract_z
_atom_site_occupancy
_atom_site_fract_symmform
Zr1 Zr 2 h 0.5000000000 0.5000000000 0.4159700000 1.0000000000 0,0,Dz
C1 C 2 g 0.0000000000 0.0000000000 0.4134500000 1.0000000000 0,0,Dz
Li1 Li 1 b 0.0000000000 0.0000000000 0.5000000000 1.0000000000 0,0,0
H1 H 1 d 0.5000000000 0.5000000000 0.5000000000 1.0000000000 0,0,0

end of cif

ZrC | LiH | ZrC - 61%

CIF file created by FINDSYM, version 7.1.3

data_findsym-output
_audit_creation_method FINDSYM

_cell_length_a 3.2168000000
_cell_length_b 3.2168000000
_cell_length_c 24.7200000000
_cell_angle_alpha 90.0000000000
_cell_angle_beta 90.0000000000
_cell_angle_gamma 90.0000000000
_cell_volume 255.7976713728

_symmetry_space_group_name_H-M "P 4/m 2/m 2/m"
_symmetry_Int_Tables_number 123
_space_group.reference_setting '123:-P 4 2'
_space_group.transform_Pp_abc a,b,c;0,0,0

loop_
_space_group_symop_id
_space_group_symop_operation_xyz
1 x,y,z
2 x,-y,-z
3 -x,y,-z
4 -x,-y,z
5 -y,-x,-z
6 -y,x,z
7 y,-x,z
8 y,x,-z
9 -x,-y,-z
10 -x,y,z
11 x,-y,z
12 x,y,-z
13 y,x,z
14 y,-x,-z
15 -y,x,-z
16 -y,-x,z

loop_
_atom_type_symbol
Zr
C
Li
H

loop_
_atom_site_label
_atom_site_type_symbol
_atom_site_symmetry_multiplicity
_atom_site_Wyckoff_symbol
_atom_site_fract_x
_atom_site_fract_y
_atom_site_fract_z
_atom_site_occupancy
_atom_site_fract_symmform
Zr1 Zr 2 h 0.5000000000 0.5000000000 0.4086900000 1.0000000000 0,0,Dz
C1 C 2 g 0.0000000000 0.0000000000 0.4133500000 1.0000000000 0,0,Dz
Li1 Li 1 b 0.0000000000 0.0000000000 0.5000000000 1.0000000000 0,0,0
H1 H 1 d 0.5000000000 0.5000000000 0.5000000000 1.0000000000 0,0,0

end of cif

$KMgF_3$ | LiH | $KMgF_3$ - 0%

CIF file created by FINDSYM, version 7.1.3

data_findsym-output
_audit_creation_method FINDSYM

_cell_length_a 3.9972000000
_cell_length_b 3.9972000000
_cell_length_c 22.2224000000
_cell_angle_alpha 90.0000000000
_cell_angle_beta 90.0000000000
_cell_angle_gamma 90.0000000000
_cell_volume 355.0607924636

_symmetry_space_group_name_H-M "P 4/m 2/m 2/m"
_symmetry_Int_Tables_number 123
_space_group.reference_setting '123:-P 4 2'
_space_group.transform_Pp_abc a,b,c;0,0,0

loop_
_space_group_symop_id
_space_group_symop_operation_xyz
1 x,y,z
2 x,-y,-z
3 -x,y,-z
4 -x,-y,z
5 -y,-x,-z
6 -y,x,z
7 y,-x,z
8 y,x,-z
9 -x,-y,-z
10 -x,y,z
11 x,-y,z
12 x,y,-z
13 y,x,z
14 y,-x,-z
15 -y,x,-z
16 -y,-x,z

```
loop_
_atom_type_symbol
K
Mg
Li
H
F

loop_
_atom_site_label
_atom_site_type_symbol
_atom_site_symmetry_multiplicity
_atom_site_Wyckoff_symbol
_atom_site_fract_x
_atom_site_fract_y
_atom_site_fract_z
_atom_site_occupancy
_atom_site_fract_symmform
K1 K 2 h 0.5000000000 0.5000000000 0.3187750000 1.0000000000 0,0,Dz
Mg1 Mg 2 g 0.0000000000 0.0000000000 0.4021700000 1.0000000000 0,0,Dz
Li1 Li 2 e 0.0000000000 0.5000000000 0.5000000000 1.0000000000 0,0,0
H1 H 1 d 0.5000000000 0.5000000000 0.5000000000 1.0000000000 0,0,0
H2 H 1 b 0.0000000000 0.0000000000 0.5000000000 1.0000000000 0,0,0
F1 F 2 g 0.0000000000 0.0000000000 0.3131200000 1.0000000000 0,0,Dz
F2 F 4 i 0.0000000000 0.5000000000 0.5939300000 1.0000000000 0,0,Dz

# end of cif
```

$KMgF_3$ | LiH | $KMgF_3$ - 25%

```
# CIF file created by FINDSYM, version 7.1.3

data_findsym-output
_audit_creation_method FINDSYM

_cell_length_a 3.9972000000
_cell_length_b 3.9972000000
_cell_length_c 22.2224000000
_cell_angle_alpha 90.0000000000
_cell_angle_beta 90.0000000000
_cell_angle_gamma 90.0000000000
_cell_volume 355.0607924636

_symmetry_space_group_name_H-M "P 4/m 2/m 2/m"
_symmetry_Int_Tables_number 123
_space_group.reference_setting '123:-P 4 2'
_space_group.transform_Pp_abc a,b,c;0,0,0

loop_
_space_group_symop_id
_space_group_symop_operation_xyz
1 x,y,z
2 x,-y,-z
3 -x,y,-z
4 -x,-y,z
5 -y,-x,-z
```

6 -y,x,z
7 y,-x,z
8 y,x,-z
9 -x,-y,-z
10 -x,y,z
11 x,-y,z
12 x,y,-z
13 y,x,z
14 y,-x,-z
15 -y,x,-z
16 -y,-x,z

loop_
_atom_type_symbol
K
Mg
Li
H
F

loop_
_atom_site_label
_atom_site_type_symbol
_atom_site_symmetry_multiplicity
_atom_site_Wyckoff_symbol
_atom_site_fract_x
_atom_site_fract_y
_atom_site_fract_z
_atom_site_occupancy
_atom_site_fract_symmform
K1 K 2 h 0.5000000000 0.5000000000 0.3020950000 1.0000000000 0,0,Dz
Mg1 Mg 2 g 0.0000000000 0.0000000000 0.3983200000 1.0000000000 0,0,Dz
Li1 Li 2 e 0.0000000000 0.5000000000 0.5000000000 1.0000000000 0,0,0
H1 H 1 d 0.5000000000 0.5000000000 0.5000000000 1.0000000000 0,0,0
H2 H 1 b 0.0000000000 0.0000000000 0.5000000000 1.0000000000 0,0,0
F1 F 2 g 0.0000000000 0.0000000000 0.3116800000 1.0000000000 0,0,Dz
F2 F 4 i 0.0000000000 0.5000000000 0.5901500000 1.0000000000 0,0,Dz

end of cif

$(KMgF_3)_2$ | LiH | $(KMgF_3)_2$ - 0%

CIF file created by FINDSYM, version 7.1.3

data_findsym-output
_audit_creation_method FINDSYM

_cell_length_a 4.0183000000
_cell_length_b 4.0183000000
_cell_length_c 32.0240000000
_cell_angle_alpha 90.0000000000
_cell_angle_beta 90.0000000000
_cell_angle_gamma 90.0000000000
_cell_volume 517.0830381174

_symmetry_space_group_name_H-M "P 4/m 2/m 2/m"
_symmetry_Int_Tables_number 123

_space_group.reference_setting '123:-P 4 2'
_space_group.transform_Pp_abc a,b,c;0,0,0

loop_ _space_group_symop_id
_space_group_symop_operation_xyz
1 x,y,z
2 x,-y,-z
3 -x,y,-z
4 -x,-y,z
5 -y,-x,-z
6 -y,x,z
7 y,-x,z
8 y,x,-z
9 -x,-y,-z
10 -x,y,z
11 x,-y,z
12 x,y,-z
13 y,x,z
14 y,-x,-z
15 -y,x,-z
16 -y,-x,z

loop_
_atom_site_label
_atom_site_type_symbol
_atom_site_symmetry_multiplicity
_atom_site_Wyckoff_symbol
_atom_site_fract_x
_atom_site_fract_y
_atom_site_fract_z
_atom_site_occupancy
_atom_site_fract_symmform
K1 K 2 g 0.00000 0.00000 0.24620 1.00000 0,0,Dz
K2 K 2 g 0.00000 0.00000 0.37258 1.00000 0,0,Dz
Mg1 Mg 2 h 0.50000 0.50000 0.30672 1.00000 0,0,Dz
Mg2 Mg 2 h 0.50000 0.50000 0.43285 1.00000 0,0,Dz
F1 F 2 h 0.50000 0.50000 0.24405 1.00000 0,0,Dz
F2 F 4 i 0.00000 0.50000 0.30762 1.00000 0,0,Dz
F3 F 2 h 0.50000 0.50000 0.37061 1.00000 0,0,Dz
F4 F 4 i 0.00000 0.50000 0.43494 1.00000 0,0,Dz
H1 H 1 b 0.00000 0.00000 0.50000 1.00000 0,0,0
H2 H 1 d 0.50000 0.50000 0.50000 1.00000 0,0,0
Li1 Li 2 e 0.00000 0.50000 0.50000 1.00000 0,0,0

end of cif

$(KMgF_3)_2$ | LiH | $(KMgF_3)_2$ - 20%

CIF file created by FINDSYM, version 7.1.3

data_findsym-output
_audit_creation_method FINDSYM

_cell_length_a 4.0183000000
_cell_length_b 4.0183000000
_cell_length_c 32.0240000000
_cell_angle_alpha 90.0000000000

_cell_angle_beta 90.0000000000
_cell_angle_gamma 90.0000000000
_cell_volume 517.0830381174

_symmetry_space_group_name_H-M "P 4/m 2/m 2/m"
_symmetry_Int_Tables_number 123
_space_group.reference_setting '123:-P 4 2'
_space_group.transform_Pp_abc a,b,c;0,0,0

loop_
_space_group_symop_id
_space_group_symop_operation_xyz
1 x,y,z
2 x,-y,-z
3 -x,y,-z
4 -x,-y,z
5 -y,-x,-z
6 -y,x,z
7 y,-x,z
8 y,x,-z
9 -x,-y,-z
10 -x,y,z
11 x,-y,z
12 x,y,-z
13 y,x,z
14 y,-x,-z
15 -y,x,-z
16 -y,-x,z

loop_
_atom_site_label
_atom_site_type_symbol
_atom_site_symmetry_multiplicity
_atom_site_Wyckoff_symbol
_atom_site_fract_x
_atom_site_fract_y
_atom_site_fract_z
_atom_site_occupancy
_atom_site_fract_symmform
K1 K 2 g 0.00000 0.00000 0.23772 1.00000 0,0,Dz
K2 K 2 g 0.00000 0.00000 0.36576 1.00000 0,0,Dz
Mg1 Mg 2 h 0.50000 0.50000 0.30273 1.00000 0,0,Dz
Mg2 Mg 2 h 0.50000 0.50000 0.43028 1.00000 0,0,Dz
F1 F 2 h 0.50000 0.50000 0.24151 1.00000 0,0,Dz
F2 F 4 i 0.00000 0.50000 0.30622 1.00000 0,0,Dz
F3 F 2 h 0.50000 0.50000 0.36973 1.00000 0,0,Dz
F4 F 4 i 0.00000 0.50000 0.43731 1.00000 0,0,Dz
H1 H 1 b 0.00000 0.00000 0.50000 1.00000 0,0,0
H2 H 1 d 0.50000 0.50000 0.50000 1.00000 0,0,0
Li1 Li 2 e 0.00000 0.50000 0.50000 1.00000 0,0,0

end of cif

$LiBaF_3$ | LiH | $LiBaF_3$ - 0%

CIF file created by FINDSYM, version 7.1.3

data_findsym-output
_audit_creation_method FINDSYM

_cell_length_a 3.9974000000
_cell_length_b 3.9974000000
_cell_length_c 30.0206000000
_cell_angle_alpha 90.0000000000
_cell_angle_beta 90.0000000000
_cell_angle_gamma 90.0000000000
_cell_volume 479.7053744593

_symmetry_space_group_name_H-M ”P 4/m 2/m 2/m”
_symmetry_Int_Tables_number 123
_space_group.reference_setting ’123:-P 4 2’
_space_group.transform_Pp_abc a,b,c;0,0,0

loop_
_space_group_symop_id
_space_group_symop_operation_xyz
1 x,y,z
2 x,-y,-z
3 -x,y,-z
4 -x,-y,z
5 -y,-x,-z
6 -y,x,z
7 y,-x,z
8 y,x,-z
9 -x,-y,-z
10 -x,y,z
11 x,-y,z
12 x,y,-z
13 y,x,z
14 y,-x,-z
15 -y,x,-z
16 -y,-x,z

loop_
_atom_type_symbol
Ba
Li
F
H

loop_
_atom_site_label
_atom_site_type_symbol
_atom_site_symmetry_multiplicity
_atom_site_Wyckoff_symbol
_atom_site_fract_x
_atom_site_fract_y
_atom_site_fract_z
_atom_site_occupancy
_atom_site_fract_symmform
Ba1 Ba 2 g 0.0000000000 0.0000000000 0.3732100000 1.0000000000 0,0,Dz
Li1 Li 2 h 0.5000000000 0.5000000000 0.4368400000 1.0000000000 0,0,Dz
Li2 Li 2 e 0.0000000000 0.5000000000 0.5000000000 1.0000000000 0,0,0
F1 F 2 h 0.5000000000 0.5000000000 0.3503900000 1.0000000000 0,0,Dz
F2 F 4 i 0.0000000000 0.5000000000 0.4267000000 1.0000000000 0,0,Dz

H1 H 1 d 0.5000000000 0.5000000000 0.5000000000 1.0000000000 0,0,0
H2 H 1 b 0.0000000000 0.0000000000 0.5000000000 1.0000000000 0,0,0

end of cif

$LiBaF_3$ | LiH | $LiBaF_3$ - 31%

CIF file created by FINDSYM, version 7.1.3

data_findsym-output
_audit_creation_method FINDSYM

_cell_length_a 3.9974000000
_cell_length_b 3.9974000000
_cell_length_c 30.0206000000
_cell_angle_alpha 90.0000000000
_cell_angle_beta 90.0000000000
_cell_angle_gamma 90.0000000000
_cell_volume 479.7053744593

_symmetry_space_group_name_H-M "P 4/m 2/m 2/m"
_symmetry_Int_Tables_number 123
_space_group.reference_setting '123:-P 4 2'
_space_group.transform_Pp_abc a,b,c;0,0,0

loop_
_space_group_symop_id
_space_group_symop_operation_xyz
1 x,y,z
2 x,-y,-z
3 -x,y,-z
4 -x,-y,z
5 -y,-x,-z
6 -y,x,z
7 y,-x,z
8 y,x,-z
9 -x,-y,-z
10 -x,y,z
11 x,-y,z
12 x,y,-z
13 y,x,z
14 y,-x,-z
15 -y,x,-z
16 -y,-x,z

loop_
_atom_type_symbol
Ba
Li
F
H

loop_
_atom_site_label
_atom_site_type_symbol
_atom_site_symmetry_multiplicity
_atom_site_Wyckoff_symbol

_atom_site_fract_x
_atom_site_fract_y
_atom_site_fract_z
_atom_site_occupancy
_atom_site_fract_symmform
Ba1 Ba 2 g 0.0000000000 0.0000000000 0.3728100000 1.0000000000 0,0,Dz
Li1 Li 2 h 0.5000000000 0.5000000000 0.4306300000 1.0000000000 0,0,Dz
Li2 Li 2 e 0.0000000000 0.5000000000 0.5000000000 1.0000000000 0,0,0
F1 F 2 h 0.5000000000 0.5000000000 0.3624900000 1.0000000000 0,0,Dz
F2 F 4 i 0.0000000000 0.5000000000 0.4314100000 1.0000000000 0,0,Dz
H1 H 1 d 0.5000000000 0.5000000000 0.5000000000 1.0000000000 0,0,0
H2 H 1 b 0.0000000000 0.0000000000 0.5000000000 1.0000000000 0,0,0

end of cif

$(LiBaF_3)_2$ | LiH | $(LiBaF_3)_2$ - 0%

CIF file created by FINDSYM, version 7.1.3

data_findsym-output
_audit_creation_method FINDSYM

_cell_length_a 4.0026000000
_cell_length_b 4.0026000000
_cell_length_c 40.5574000000
_cell_angle_alpha 90.0000000000
_cell_angle_beta 90.0000000000
_cell_angle_gamma 90.0000000000
_cell_volume 649.7622680880

_symmetry_space_group_name_H-M "P 4/m 2/m 2/m"
_symmetry_Int_Tables_number 123
_space_group.reference_setting '123:-P 4 2'
_space_group.transform_Pp_abc a,b,c;0,0,0

loop_
_space_group_symop_id
_space_group_symop_operation_xyz
1 x,y,z
2 x,-y,-z
3 -x,y,-z
4 -x,-y,z
5 -y,-x,-z
6 -y,x,z
7 y,-x,z
8 y,x,-z
9 -x,-y,-z
10 -x,y,z
11 x,-y,z
12 x,y,-z
13 y,x,z
14 y,-x,-z
15 -y,x,-z
16 -y,-x,z

loop_
_atom_site_label

_atom_site_type_symbol
_atom_site_symmetry_multiplicity
_atom_site_Wyckoff_symbol
_atom_site_fract_x
_atom_site_fract_y
_atom_site_fract_z
_atom_site_occupancy
_atom_site_fract_symmform
Ba1 Ba 2 g 0.00000 0.00000 0.70507 1.00000 0,0,Dz
Ba2 Ba 2 g 0.00000 0.00000 0.59385 1.00000 0,0,Dz
Li1 Li 2 e 0.00000 0.50000 0.50000 1.00000 0,0,0
Li2 Li 2 h 0.50000 0.50000 0.65378 1.00000 0,0,Dz
Li3 Li 2 h 0.50000 0.50000 0.54667 1.00000 0,0,Dz
F1 F 4 i 0.00000 0.50000 0.66444 1.00000 0,0,Dz
F2 F 2 h 0.50000 0.50000 0.60843 1.00000 0,0,Dz
F3 F 4 i 0.00000 0.50000 0.55423 1.00000 0,0,Dz
F4 F 2 h 0.50000 0.50000 0.27728 1.00000 0,0,Dz
H1 H 1 d 0.50000 0.50000 0.50000 1.00000 0,0,0
H2 H 1 b 0.00000 0.00000 0.50000 1.00000 0,0,0

end of cif

$(LiBaF_3)_2$ | LiH | $(LiBaF_3)_2$ - 31%

CIF file created by FINDSYM, version 7.1.3

data_findsym-output
_audit_creation_method FINDSYM

_cell_length_a 4.0026000000
_cell_length_b 4.0026000000
_cell_length_c 40.5574000000
_cell_angle_alpha 90.0000000000
_cell_angle_beta 90.0000000000
_cell_angle_gamma 90.0000000000
_cell_volume 649.7622680880

_symmetry_space_group_name_H-M "P 4/m 2/m 2/m"
_symmetry_Int_Tables_number 123
_space_group.reference_setting '123:-P 4 2'
_space_group.transform_Pp_abc a,b,c;0,0,0

loop_
_space_group_symop_id
_space_group_symop_operation_xyz
1 x,y,z
2 x,-y,-z
3 -x,y,-z
4 -x,-y,z
5 -y,-x,-z
6 -y,x,z
7 y,-x,z
8 y,x,-z
9 -x,-y,-z
10 -x,y,z
11 x,-y,z
12 x,y,-z

13 y,x,z
14 y,-x,-z
15 -y,x,-z
16 -y,-x,z

loop_
_atom_site_label
_atom_site_type_symbol
_atom_site_symmetry_multiplicity
_atom_site_Wyckoff_symbol
_atom_site_fract_x
_atom_site_fract_y
_atom_site_fract_z
_atom_site_occupancy
_atom_site_fract_symmform
Ba1 Ba 2 g 0.00000 0.00000 0.69780 1.00000 0,0,Dz
Ba2 Ba 2 g 0.00000 0.00000 0.59385 1.00000 0,0,Dz
Li1 Li 2 e 0.00000 0.50000 0.50000 1.00000 0,0,0
Li2 Li 2 h 0.50000 0.50000 0.64874 1.00000 0,0,Dz
Li3 Li 2 h 0.50000 0.50000 0.55037 1.00000 0,0,Dz
F1 F 4 i 0.00000 0.50000 0.65379 1.00000 0,0,Dz
F2 F 2 h 0.50000 0.50000 0.60088 1.00000 0,0,Dz
F3 F 4 i 0.00000 0.50000 0.55013 1.00000 0,0,Dz
F4 F 2 h 0.50000 0.50000 0.29284 1.00000 0,0,Dz
H1 H 1 d 0.50000 0.50000 0.50000 1.00000 0,0,0
H2 H 1 b 0.00000 0.00000 0.50000 1.00000 0,0,0

end of cif

$RbMgF_3$ | LiH | $RbMgF_3$ - 0%

CIF file created by FINDSYM, version 7.1.3

data_findsym-output
_audit_creation_method FINDSYM

_cell_length_a 4.0514000000
_cell_length_b 4.0514000000
_cell_length_c 22.2224000000
_cell_angle_alpha 90.0000000000
_cell_angle_beta 90.0000000000
_cell_angle_gamma 90.0000000000
_cell_volume 364.7549615719

_symmetry_space_group_name_H-M "P 4/m 2/m 2/m"
_symmetry_Int_Tables_number 123
_space_group.reference_setting '123:-P 4 2'
_space_group.transform_Pp_abc a,b,c;0,0,0

loop_
_space_group_symop_id
_space_group_symop_operation_xyz
1 x,y,z
2 x,-y,-z
3 -x,y,-z
4 -x,-y,z
5 -y,-x,-z

6 -y,x,z
7 y,-x,z
8 y,x,-z
9 -x,-y,-z
10 -x,y,z
11 x,-y,z
12 x,y,-z
13 y,x,z
14 y,-x,-z
15 -y,x,-z
16 -y,-x,z

loop_
_atom_site_label
_atom_site_type_symbol
_atom_site_symmetry_multiplicity
_atom_site_Wyckoff_symbol
_atom_site_fract_x
_atom_site_fract_y
_atom_site_fract_z
_atom_site_occupancy
_atom_site_fract_symmform
Rb1 Rb 2 h 0.50000 0.50000 0.31335 1.00000 0,0,Dz
Mg1 Mg 2 g 0.00000 0.00000 0.40333 1.00000 0,0,Dz
Li1 Li 2 e 0.00000 0.50000 0.50000 1.00000 0,0,0
H1 H 1 d 0.50000 0.50000 0.50000 1.00000 0,0,0
H2 H 1 b 0.00000 0.00000 0.50000 1.00000 0,0,0
F1 F 2 g 0.00000 0.00000 0.31379 1.00000 0,0,Dz
F2 F 4 i 0.00000 0.50000 0.59299 1.00000 0,0,Dz

end of cif

$RbMgF_3$ | LiH | $RbMgF_3$ - 25%

CIF file created by FINDSYM, version 7.1.3

data_findsym-output
_audit_creation_method FINDSYM

_cell_length_a 4.0514000000
_cell_length_b 4.0514000000
_cell_length_c 22.2224000000
_cell_angle_alpha 90.0000000000
_cell_angle_beta 90.0000000000
_cell_angle_gamma 90.0000000000
_cell_volume 364.7549615719

_symmetry_space_group_name_H-M "P 4/m 2/m 2/m"
_symmetry_Int_Tables_number 123
_space_group.reference_setting '123:-P 4 2'
_space_group.transform_Pp_abc a,b,c;0,0,0

loop_
_space_group_symop_id
_space_group_symop_operation_xyz
1 x,y,z
2 x,-y,-z

3 -x,y,-z
4 -x,-y,z
5 -y,-x,-z
6 -y,x,z
7 y,-x,z
8 y,x,-z
9 -x,-y,-z
10 -x,y,z
11 x,-y,z
12 x,y,-z
13 y,x,z
14 y,-x,-z
15 -y,x,-z
16 -y,-x,z

loop_
_atom_type_symbol
Rb
Mg
Li
H
F

loop_
_atom_site_label
_atom_site_type_symbol
_atom_site_symmetry_multiplicity
_atom_site_Wyckoff_symbol
_atom_site_fract_x
_atom_site_fract_y
_atom_site_fract_z
_atom_site_occupancy
_atom_site_fract_symmform
Rb1 Rb 2 h 0.5000000000 0.5000000000 0.2985000000 1.0000000000 0,0,Dz
Mg1 Mg 2 g 0.0000000000 0.0000000000 0.3998800000 1.0000000000 0,0,Dz
Li1 Li 2 e 0.0000000000 0.5000000000 0.5000000000 1.0000000000 0,0,0
H1 H 1 d 0.5000000000 0.5000000000 0.5000000000 1.0000000000 0,0,0
H2 H 1 b 0.0000000000 0.0000000000 0.5000000000 1.0000000000 0,0,0
F1 F 2 g 0.0000000000 0.0000000000 0.3130800000 1.0000000000 0,0,Dz
F2 F 4 i 0.0000000000 0.5000000000 0.5891550000 1.0000000000 0,0,Dz

end of cif

$(RbMgF_3)_2$ | LiH | $(RbMgF_3)_2$ - 0%

CIF file created by FINDSYM, version 7.1.3

data_findsym-output
_audit_creation_method FINDSYM

_cell_length_a 4.0813000000
_cell_length_b 4.0813000000
_cell_length_c 32.0800000000
_cell_angle_alpha 90.0000000000
_cell_angle_beta 90.0000000000
_cell_angle_gamma 90.0000000000
_cell_volume 534.3568708552

_symmetry_space_group_name_H-M "P 4/m 2/m 2/m"
_symmetry_Int_Tables_number 123
_space_group.reference_setting '123:-P 4 2'
_space_group.transform_Pp_abc a,b,c;0,0,0

loop_
_space_group_symop_id
_space_group_symop_operation_xyz
1 x,y,z
2 x,-y,-z
3 -x,y,-z
4 -x,-y,z
5 -y,-x,-z
6 -y,x,z
7 y,-x,z
8 y,x,-z
9 -x,-y,-z
10 -x,y,z
11 x,-y,z
12 x,y,-z
13 y,x,z
14 y,-x,-z
15 -y,x,-z
16 -y,-x,z

loop_
_atom_site_label
_atom_site_type_symbol
_atom_site_symmetry_multiplicity
_atom_site_Wyckoff_symbol
_atom_site_fract_x
_atom_site_fract_y
_atom_site_fract_z
_atom_site_occupancy
_atom_site_fract_symmform
Rb1 Rb 2 g 0.00000 0.00000 0.24014 1.00000 0,0,Dz
Rb2 Rb 2 g 0.00000 0.00000 0.37171 1.00000 0,0,Dz
Mg1 Mg 2 h 0.50000 0.50000 0.30513 1.00000 0,0,Dz
Mg2 Mg 2 h 0.50000 0.50000 0.43329 1.00000 0,0,Dz
F1 F 2 h 0.50000 0.50000 0.24229 1.00000 0,0,Dz
F2 F 4 i 0.00000 0.50000 0.30544 1.00000 0,0,Dz
F3 F 2 h 0.50000 0.50000 0.37008 1.00000 0,0,Dz
F4 F 4 i 0.00000 0.50000 0.43587 1.00000 0,0,Dz
H1 H 1 b 0.00000 0.00000 0.50000 1.00000 0,0,0
H2 H 1 d 0.50000 0.50000 0.50000 1.00000 0,0,0
Li1 Li 2 e 0.00000 0.50000 0.50000 1.00000 0,0,0

end of cif

$(RbMgF_3)_2$ | LiH | $(RbMgF_3)_2$ - 27%

CIF file created by FINDSYM, version 7.1.3

data_findsym-output
_audit_creation_method FINDSYM

```
_cell_length_a 4.0813000000
_cell_length_b 4.0813000000
_cell_length_c 32.0800000000
_cell_angle_alpha 90.0000000000
_cell_angle_beta 90.0000000000
_cell_angle_gamma 90.0000000000
_cell_volume 534.3568708552

_symmetry_space_group_name_H-M "P 4/m 2/m 2/m"
_symmetry_Int_Tables_number 123
_space_group.reference_setting '123:-P 4 2'
_space_group.transform_Pp_abc a,b,c;0,0,0

loop_
_space_group_symop_id
_space_group_symop_operation_xyz
1 x,y,z
2 x,-y,-z
3 -x,y,-z
4 -x,-y,z
5 -y,-x,-z
6 -y,x,z
7 y,-x,z
8 y,x,-z
9 -x,-y,-z
10 -x,y,z
11 x,-y,z
12 x,y,-z
13 y,x,z
14 y,-x,-z
15 -y,x,-z
16 -y,-x,z

loop_
_atom_site_label
_atom_site_type_symbol
_atom_site_symmetry_multiplicity
_atom_site_Wyckoff_symbol
_atom_site_fract_x
_atom_site_fract_y
_atom_site_fract_z
_atom_site_occupancy
_atom_site_fract_symmform
Rb1 Rb 2 g 0.00000 0.00000 0.22845 1.00000 0,0,Dz
Rb2 Rb 2 g 0.00000 0.00000 0.36445 1.00000 0,0,Dz
Mg1 Mg 2 h 0.50000 0.50000 0.29874 1.00000 0,0,Dz
Mg2 Mg 2 h 0.50000 0.50000 0.43010 1.00000 0,0,Dz
F1 F 2 h 0.50000 0.50000 0.23794 1.00000 0,0,Dz
F2 F 4 i 0.00000 0.50000 0.30286 1.00000 0,0,Dz
F3 F 2 h 0.50000 0.50000 0.36977 1.00000 0,0,Dz
F4 F 4 i 0.00000 0.50000 0.43897 1.00000 0,0,Dz
H1 H 1 b 0.00000 0.00000 0.50000 1.00000 0,0,0
H2 H 1 d 0.50000 0.50000 0.50000 1.00000 0,0,0
Li1 Li 2 e 0.00000 0.50000 0.50000 1.00000 0,0,0

# end of cif
```

[1] P. B. Allen and R. C. Dynes. Transition temperature of strong-coupled superconductors reanalyzed. *Phys. Rev. B*, 12:905–922, Aug 1975.
[2] Stefano Baroni, Stefano de Gironcoli, Andrea Dal Corso, and Paolo Giannozzi. Phonons and related crystal properties from density-functional perturbation theory. *Rev. Mod. Phys.*, 73:515–562, Jul 2001.
[3] P Giannozzi, O Andreussi, T Brumme, O Bunau, M Buongiorno Nardelli, M Calandra, R Car, C Cavazzoni, D Ceresoli, M Cococcioni, N Colonna, I Carnimeo, A Dal Corso, S de Gironcoli, P Delugas, R A DiStasio, A Ferretti, A Floris, G Fratesi, G Fugallo, R Gebauer, U Gerstmann, F Giustino, T Gorni, J Jia, M Kawamura, H-Y Ko, A Kokalj, E Küçükbenli, M Lazzeri, M Marsili, N Marzari, F Mauri, N L Nguyen, H-V Nguyen, A Otero de-la Roza, L Paulatto, S Poncé, D Rocca, R Sabatini, B Santra, M Schlipf, A P Seitsonen, A Smogunov, I Timrov, T Thonhauser, P Umari, N Vast, X Wu, and S Baroni. Advanced capabilities for materials modelling with quantum espresso. *Journal of Physics: Condensed Matter*, 29(46):465901, oct 2017.
[4] Paolo Giannozzi, Stefano Baroni, Nicola Bonini, Matteo Calandra, Roberto Car, Carlo Cavazzoni, Davide Ceresoli, Guido L Chiarotti, Matteo Cococcioni, Ismaila Dabo, Andrea Dal Corso, Stefano de Gironcoli, Stefano Fabris, Guido Fratesi, Ralph Gebauer, Uwe Gerstmann, Christos Gougoussis, Anton Kokalj, Michele Lazzeri, Layla Martin-Samos, Nicola Marzari, Francesco Mauri, Riccardo Mazzarello, Stefano Paolini, Alfredo Pasquarello, Lorenzo Paulatto, Carlo Sbraccia, Sandro Scandolo, Gabriele Sclauzero, Ari P Seitsonen, Alexander Smogunov, Paolo Umari, and Renata M Wentzcovitch. QUANTUM ESPRESSO: a modular and open-source software project for quantum simulations of materials. *Journal of Physics: Condensed Matter*, 21(39):395502, sep 2009.
[5] W. L. McMillan. Transition temperature of strong-coupled superconductors. *Phys. Rev.*, 167:331–344, Mar 1968.
[6] M. Methfessel and A. T. Paxton. High-precision sampling for Brillouin-zone integration in metals. *Phys. Rev. B*, 40:3616–3621, Aug 1989.
[7] Hendrik J. Monkhorst and James D. Pack. Special points for Brillouin-zone integrations. *Phys. Rev. B*, 13:5188–5192, Jun 1976.
[8] John P. Perdew, Kieron Burke, and Matthias Ernzerhof. Generalized Gradient Approximation Made Simple. *Phys. Rev. Lett.*, 77:3865–3868, Oct 1996.
[9] M.J. van Setten, M. Giantomassi, E. Bousquet, M.J. Verstraete, D.R. Hamann, X. Gonze, and G.-M. Rignanese. The PseudoDojo: Training and grading a 85 element optimized norm-conserving pseudopotential table. *Computer Physics Communications*, 226:39–54, 2018.
[10] Małgorzata Wierzbowska, Stefano de Gironcoli, and Paolo Giannozzi. Origins of low- and high-pressure discontinuities of T_c in niobium. *arXiv: Superconductivity*, 2005.
